# Conserved crosstalk between histone deacetylation and H3K79 methylation generates DOT1L‐dose dependency in HDAC1‐deficient thymic lymphoma

**DOI:** 10.15252/embj.2019101564

**Published:** 2019-06-17

**Authors:** Hanneke Vlaming, Chelsea M McLean, Tessy Korthout, Mir Farshid Alemdehy, Sjoerd Hendriks, Cesare Lancini, Sander Palit, Sjoerd Klarenbeek, Eliza Mari Kwesi‐Maliepaard, Thom M Molenaar, Liesbeth Hoekman, Thierry T Schmidlin, AF Maarten Altelaar, Tibor van Welsem, Jan‐Hermen Dannenberg, Heinz Jacobs, Fred van Leeuwen

**Affiliations:** ^1^ Division of Gene Regulation Netherlands Cancer Institute Amsterdam The Netherlands; ^2^ Division of Tumor Biology & Immunology Netherlands Cancer Institute Amsterdam The Netherlands; ^3^ Experimental Animal Pathology Netherlands Cancer Institute Amsterdam The Netherlands; ^4^ Biomolecular Mass Spectrometry and Proteomics Bijvoet Center for Biomolecular Research Utrecht Institute for Pharmaceutical Sciences Utrecht University and Netherlands Proteomics Centre Utrecht The Netherlands; ^5^ Proteomics Facility Netherlands Cancer Institute Amsterdam The Netherlands; ^6^ Present address: Department of Biological Chemistry and Molecular Pharmacology Harvard Medical School Boston MA USA; ^7^ Present address: Genmab B.V. Antibody Sciences Utrecht The Netherlands

**Keywords:** Chromatin, H3K79 methylation, histone acetylation, histone ubiquitination, lymphoma, Cancer, Chromatin, Epigenetics, Genomics & Functional Genomics

## Abstract

DOT1L methylates histone H3K79 and is aberrantly regulated in MLL‐rearranged leukemia. Inhibitors have been developed to target DOT1L activity in leukemia, but cellular mechanisms that regulate DOT1L are still poorly understood. We have identified the histone deacetylase Rpd3 as a negative regulator of budding yeast Dot1. At its target genes, the transcriptional repressor Rpd3 restricts H3K79 methylation, explaining the absence of H3K79me3 at a subset of genes in the yeast genome. Similar to the crosstalk in yeast, inactivation of the murine Rpd3 homolog HDAC1 in thymocytes led to an increase in H3K79 methylation. Thymic lymphomas that arise upon genetic deletion of *Hdac1* retained the increased H3K79 methylation and were sensitive to reduced DOT1L dosage. Furthermore, cell lines derived from *Hdac1* ^*Δ/Δ*^ thymic lymphomas were sensitive to a DOT1L inhibitor, which induced apoptosis. In summary, we identified an evolutionarily conserved crosstalk between HDAC1 and DOT1L with impact in murine thymic lymphoma development.

## Introduction

Aberrant histone modification patterns have been observed in many diseases, and this deregulation of chromatin can play a causative role in disease. Since epigenetic alterations are, in principle, reversible in nature, histone (de)modifiers are attractive therapeutic targets (Brien *et al*, 2016; Jones *et al*, 2016; Shortt *et al*, 2017). Several epigenetic drugs are currently in the clinic or in clinical trials, but for many of the drug targets, we are only beginning to understand their cellular regulation.

The histone H3K79 methyltransferase DOT1L (KMT4; Dot1 in yeast) is an epigenetic enzyme for which inhibitors are in clinical development for the treatment of MLL‐rearranged (MLL‐r) leukemia (Stein & Tallman, 2016). In MLL‐r leukemia, DOT1L recruitment to MLL target genes, such as the HoxA cluster, leads to aberrant H3K79 methylation and increased transcription (reviewed in Vlaming & Van Leeuwen, 2016). Although the DOT1L inhibitor Pinometostat (EPZ‐5676) has shown promising results in the laboratory and is currently in clinical development (Bernt *et al*, 2011; Daigle *et al*, 2013; Waters *et al*, 2015; Stein & Tallman, 2016; Stein *et al*, 2018), the cellular mechanisms and consequences of DOT1L deregulation are only just being uncovered (Vlaming & Van Leeuwen, 2016).

An important mechanism of regulation is the trans‐histone crosstalk between monoubiquitination of the C‐terminus of histone H2B (H2Bub) at lysine 120 (123 in yeast) and methylation of histone H3K79 (Zhang *et al*, 2015). The addition of a ubiquitin peptide to the nucleosome at this position occurs in a co‐transcriptional manner and promotes the activity of Dot1/DOT1L, possibly by activation of DOT1L or coaching it toward H3K79 and thereby increasing the chance of a productive encounter (Vlaming *et al*, 2014; Zhou *et al*, 2016). Another mechanism of regulation is mediated by the direct interactions of DOT1L with central transcription elongation proteins (reviewed in Vlaming & Van Leeuwen, 2016). These interactions target DOT1L to transcribed chromatin and provide an explanation for the aberrant recruitment of DOT1L by oncogenic MLL fusion proteins (Deshpande *et al*, 2014; Li *et al*, 2014; Chen *et al*, 2015; Kuntimaddi *et al*, 2015; Wood *et al*, 2018). Further characterizing the regulatory network of DOT1L could lead to the identification of alternative drug targets for diseases in which DOT1L is critical and provide alternative strategies in case of resistance to treatment with DOT1L inhibitors (Campbell *et al*, 2017).

In a previous study, we presented a ChIP‐barcode‐seq screen (Epi‐ID) identifying novel regulators of H3K79 methylation in yeast (Vlaming *et al*, 2016). The Rpd3‐large (Rpd3L) complex was identified as an enriched complex among the candidate negative regulators of H3K79 methylation of a barcoded reporter gene. Rpd3 is a class I histone deacetylase (HDAC) that removes acetyl groups of histones, as well as numerous non‐histone proteins, and is generally associated with transcriptional repression (Yang & Seto, 2008). Several inhibitors of mammalian HDACs have been approved for the treatment of cutaneous T‐cell lymphoma and other hematologic malignancies, while others are currently being tested in clinical trials (West & Johnstone, 2014). HDAC1 and HDAC2, prominent members of the class I HDACs, are found in the repressive Sin3, NuRD, and CoREST complexes (Yang & Seto, 2008). Loss or inhibition of HDAC1/Rpd3 leads to increased histone acetylation, which in turn can lead to increased expression of target genes and cryptic transcripts (Carrozza *et al*, 2005; Joshi & Struhl, 2005; Li *et al*, 2007; Rando & Winston, 2012; Brocks *et al*, 2017; McDaniel & Strahl, 2017).

Here, we demonstrate that Rpd3 restricts H3K79 methylation at its target genes. Most euchromatic genes in the yeast genome are marked by high levels of H3K79me3. We observed that a subset of the genes that do not follow this pattern has lower H3K79me3 levels due to the action of the Rpd3L complex, which deacetylates its targets and imposes strong transcriptional repression and absence of H2Bub1. Importantly, the Rpd3‐Dot1 crosstalk is conserved in mammals: Genetic ablation of *Hdac1* in murine thymocytes also leads to an increase in H3K79 methylation *in vivo*. High H3K79me is maintained in the lymphomas these mice develop, and a reduction in DOT1L activity by heterozygous deletion of *Dot1L* reduces tumor burden, an effect that was not observed upon homozygous deletion of *Dot1L*. Furthermore, DOT1L inhibitors induce apoptosis in *Hdac1*‐deficient but not *Hdac1*‐proficient thymic lymphoma cell lines, suggesting a DOT1L‐dose dependence. Taken together, our studies reveal a new, evolutionarily conserved mechanism of H3K79me regulation by Rpd3/HDAC1 with relevance for cancer development.

## Results

### Identification of the Rpd3L complex as a negative regulator of H3K79 methylation

**Figure 1:**
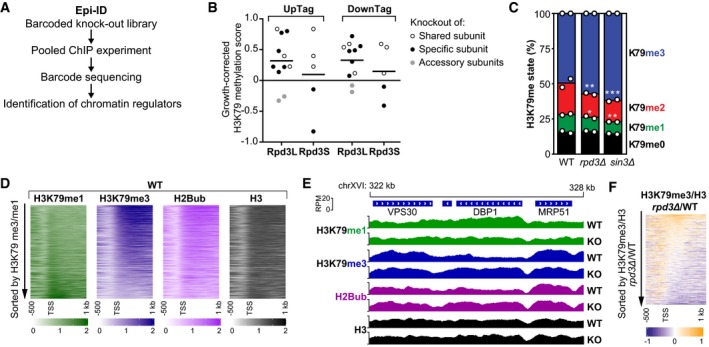
Rpd3 and other members of the Rpd3L complex negatively regulate H3K79 methylation Schematic overview of the Epi‐ID strategy.Epi‐ID H3K79 methylation scores of the deletion mutants of members of the Rpd3L and Rpd3S complexes, calculated as described in Appendix Supplementary Methods, where 0 means a wild‐type H3K79me level (log2 scale). The gray dots indicate accessory subunits. UpTag and DownTag are barcode reporters in a promoter and terminator context, respectively. Data were obtained on all Rpd3L/Rpd3S subunits except Sds3.Mass spectrometry analysis of H3K79 methylation in wild‐type and mutant strains. Mean and individual data points of two biological replicates. *P < 0.05, **P < 0.01, and ***P < 0.001 by two‐way ANOVA, comparison to wild type.Heatmaps of H3K79me1, H3K79me3, H2Bub, and H3 in wild‐type cells, aligned on the TSS. Genes were sorted based on the average H3K79me3/H3K79me1 ratio in the first 500 bp.Snapshot of depth‐normalized ChIP‐seq data tracks from wild‐type and *rpd3Δ* strains showing 6 kb surrounding the *DBP1* ORF, which is the top gene in the heatmap in panel (F). All tracks have the same y‐axis (0–20 rpm). A snapshot of another top‐regulated gene is shown in Fig EV1D.Heatmap of the H3K79me3/H3 change in *rpd3Δ* versus wild‐type cells, aligned on the TSS. Genes were sorted based on the average ratio in the first 500 bp. Source data are available online for this figure.

**Figure EV1:**
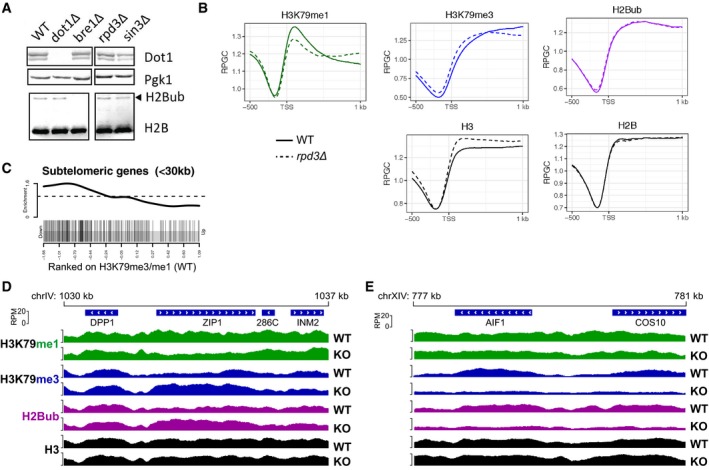
Rpd3 and Sin3 negatively regulate H3K79 methylation AImmunoblots show that deletion of *RPD3* or *SIN3* does not lead to a detectable increase in global H2Bub or Dot1 protein levels.BMetagene plots of H3K79me1, H3K79me3, H2Bub, H3, and H2B in *rpd3Δ* and wild‐type strains.CGene set enrichment analysis shows that subtelomeric genes (< 30 kb of telomeres) are enriched among genes with low H3K79 methylation (measured by the average H3K79me3/H3K79me1 ratio in the first 500 bp of the ORF).D–ESnapshots of depth‐normalized ChIP‐seq data tracks from wild‐type and *rpd3Δ* strains showing 7 kb surrounding meiotic gene *ZIP1* (D) and subtelomeric genes *AIF1* and *COS10* (E). All tracks have the same y‐axis (0–20 rpm), which, for comparison, is also the same scale as in Fig 1E. Source data are available online for this figure.

We recently reported a systematic screening strategy called Epi‐ID to identify regulators of H3K79 methylation (Vlaming *et al*, 2016). In that screen, relative H3K79 methylation (H3K79me) levels at two DNA barcodes (UpTag and DownTag) flanking a reporter gene were measured in a genome‐wide library of barcoded deletion mutants, thus testing thousands of genes for H3K79me regulator activity at these loci (Fig 1A). Since higher Dot1 activity in yeast leads to a shift from lower (me1) to higher (me3) methylation states (Frederiks *et al*, 2008), the H3K79me3 over H3K79me1 ratio was used as a measure for Dot1 activity. A growth‐corrected H3K79me score was calculated to account for the effect of growth on H3K79 methylation, and groups of positive and negative candidate regulators were identified (Vlaming *et al*, 2016). Components of the Rpd3L complex were enriched among candidate negative regulators (10‐fold over‐representation, *P* = 1.2E‐4; Vlaming *et al*, 2016). The histone deacetylase Rpd3 is found in two complexes, the large Rpd3L complex and the small Rpd3S complex, which also share the subunits Sin3 and Ume1 (Carrozza *et al*, 2005; Keogh *et al*, 2005). A closer inspection of the Rpd3 complexes revealed that deletion of Rpd3L subunits resulted in an increase in H3K79 methylation on both the UpTag and the DownTag (promoter and terminator context, respectively; Fig 1B), with the exception of two accessory subunits that play peripheral roles (Lenstra *et al*, 2011). Deletion of the two Rpd3S‐specific subunits did not lead to an increase in H3K79me (Fig 1B), which is consistent with Rpd3S binding and acting on coding sequences (Drouin *et al*, 2010) and thus away from the intergenic barcodes. To validate the effect on a global scale, we performed targeted mass spectrometry analysis to determine the relative levels of the different H3K79me states (me0 to me3) in *rpd3Δ* and *sin3Δ* strains. On bulk histones, these strains showed an increase in H3K79me (increase in H3K79me3 at the cost of lower methylation states; Fig 1C). The H3K79me increase was not caused by an increase in Dot1 protein (Fig EV1A) or mRNA expression (Kemmeren *et al*, 2014). Thus, although these regulators were identified using only two 20‐base‐pair barcodes to read out H3K79me levels at a reporter locus, their effects could be validated globally.

### Rpd3 represses H3K79 methylation at the 5′ ends of a subset of genes

We next asked at which regions Rpd3 and Sin3 regulate H3K79 methylation in yeast, other than the barcoded reporter gene. To address this, we performed ChIP‐seq analysis for H3K79me1, H3K79me3, and H3 in wild‐type and *rpd3Δ* strains. In addition, we included ChIP‐seq for H2B and H2Bub using a site‐specific antibody that we recently developed (Van Welsem *et al*, 2018). First, we considered the patterns in the wild‐type strain. Both the coverage at one representative locus and across all genes in a heatmap showed that H3K79me3 is predominantly present throughout coding sequences of most genes, where H2Bub is also high, as reported previously (Figs 1D and E, and EV1B; Schulze *et al*, 2009; Magraner‐Pardo *et al*, 2014; Weiner *et al*, 2015; Sadeh *et al*, 2016). In contrast, H3K79me1 was found in transcribed as well as intergenic regions (Figs 1D and EV1B). This is consistent with published ChIP‐seq data and our previous ChIP‐qPCR results (Weiner *et al*, 2015; Vlaming *et al*, 2016). In agreement with the distributive mechanism of methylation of Dot1 (Frederiks *et al*, 2008; De Vos *et al*, 2011), H3K79me1 and H3K79me3 anti‐correlated, and H3K79me1 over the gene body was found on the minority of genes that lacked H3K79me3 and H2Bub (Fig 1D). Among these low H3K79me3, high H3K79me1 genes were subtelomeric genes, where the SIR silencing complex competes with Dot1 for binding to nucleosomes and H2Bub levels are kept low by the deubiquitinating enzyme Ubp10 (Gardner *et al*, 2005; Emre *et al*, 2005; Gartenberg & Smith, 2016; Kueng *et al*, 2013; Fig EV1C and E).

We then compared the patterns in wild‐type versus *rpd3Δ* mutant strains. In metagene plots, the mutant showed a decrease in H3K79me1 and an increase in H3K79me3 just after the transcription start site (TSS; Fig EV1B), suggesting that in this region Rpd3 suppresses the transition from lower to higher H3K79me states. To assess whether the changes observed in the metagene plots were explained by a modest effect on H3K79me at all genes or a stronger effect at a subset of genes, we determined the H3‐normalized H3K79me3 level in the first 500 bp of each gene and ranked the genes based on the change in H3K79me3 upon loss of Rpd3. A heatmap of H3K79me3 changes by this ranking showed that the absence of Rpd3 leads to an increase in H3K79me3 at a subset of genes (Fig 1F).

### Rpd3 represses H3K79me at its target genes

**Figure 2:**
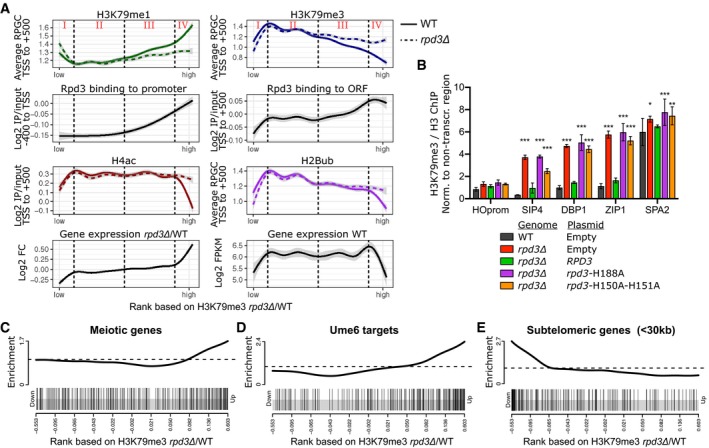
Rpd3 represses transcription, H2B ubiquitination, and H3K79 methylation at its target sites AChIP‐seq and RNA‐seq data for genes ranked on H3K79me3/H3 in *rpd3Δ*/WT, smoothed using locally weighted regression. The gray band around the line shows the 95% confidence interval. Vertical dashed lines separate 4 groups with distinct changes upon *RPD3* deletion. ChIP‐seq data of H3K79me1, H3K79me3, and H2Bub were generated in this study (plotted is the average coverage in reads per genomic content, RPGC), Rpd3 binding, H4ac, and WT gene expression data were from McKnight *et al* (2015), and the relative expression in *rpd3Δ*/WT was from Kemmeren *et al* (2014).BH3K79me3/H3 ChIP‐qPCR efficiencies (relative to a non‐transcribed region, which was unaffected by *RPD3* deletion) in wild‐type and in *rpd3Δ* cells harboring empty or *RPD3*‐encoding CEN plasmids. The H188A and H150A‐H151A mutations have previously been shown to abrogate catalytic activity (Kadosh & Struhl, 1998). Error bars indicate standard deviation of three biological replicates. *P < 0.05, **P < 0.01, and ***P < 0.001 by two‐way ANOVA, comparison to wild type.C–EGene set enrichment analysis on genes ranked on H3K79me3/H3 in *rpd3Δ*/WT; all genes have been ranked, and the ranks of the genes in the subsets are indicated by vertical lines. Meiotic (C) and Ume6‐bound (D) genes are enriched among the genes at which Rpd3 represses H3K79 methylation, and subtelomeric genes (<30 kb of telomere) (E) are enriched among genes at which H3K79 methylation is decreased in *rpd3Δ* cells. Source data are available online for this figure.

**Figure EV2:**
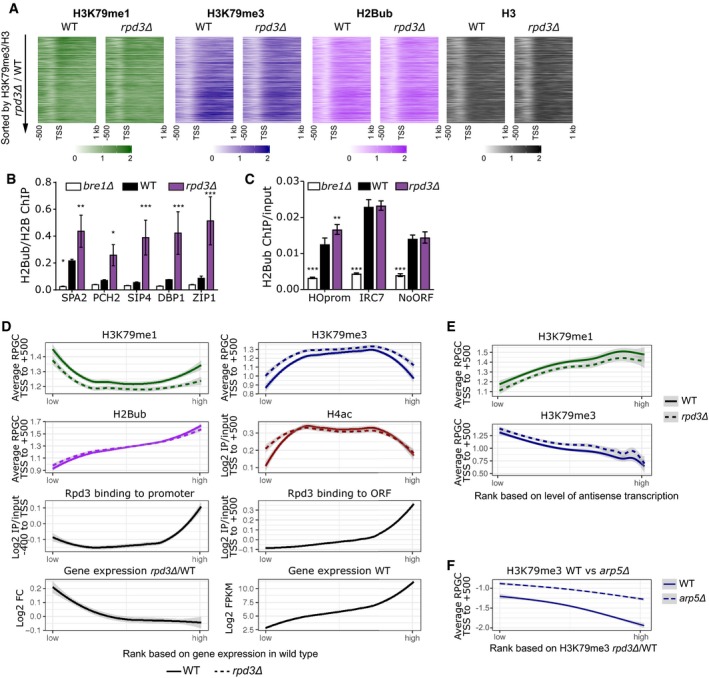
ChIP‐seq and ChIP‐qPCR data from WT vs *rpd3Δ* cells, and the relation between transcription and histone modifications Heatmaps of H3K79me1, H3K79me3, H2Bub, and H3 sorted on H3K79me3/H3 *rpd3Δ*/WT.H2Bub ChIP‐qPCR (H2Bub/H2B) at 5′ ends of indicated genes in wild‐type and *rpd3Δ* cells, with *bre1Δ* cells serving as a negative control. Error bars indicate standard deviation of three biological replicates. *P < 0.05, **P < 0.01, and ***P < 0.001 by two‐way ANOVA, comparison to wild type.H2Bub ChIP‐qPCR (H2Bub/input) at the subtelomeric IRC7 gene, the promoter of the barcoded HO locus, and a non‐transcribed region (NoORF) (Imbeault *et al*, 2008; Verzijlbergen *et al*, 2010). Error bars indicate standard deviation of three biological replicates. *P < 0.05, **P < 0.01, and ***P < 0.001 by two‐way ANOVA, comparison to wild type.ChIP‐seq and RNA‐seq data per gene (same data as in Fig 2A) ranked on gene expression level in wild‐type cells, smoothed using locally weighted regression. The shaded band around the line shows the 95% confidence interval.Similar to panel (D), but using a ranking based on the level of antisense transcription per gene in wild‐type cells as calculated by Brown *et al* (2018) using data from Churchman and Weissman (2011).H3K79me3 ChIP‐seq data in gene promoters (−400 to TSS, where the Arp5 effect is maximal) in wild‐type and *arp5Δ* cells (Xue *et al*, 2015), ranked by the effect Rpd3 has on H3K79me3 on each gene (same ranking as in Fig 2A). Source data are available online for this figure.

To characterize the genes at which H3K79me is regulated, we calculated the levels of H3K79me1 and H3K79me3 per gene in the same 500‐bp window and plotted values in the rank order of H3K79me3 changes described above, using locally weighted regression (Fig 2A; corresponding heatmaps can be found in Fig EV2A). Inspection of these plots revealed that the ORFs on which H3K79me3 was increased in the *rpd3Δ* mutant showed a simultaneous decrease in H3K79me1 (groups III–IV; Fig 2A). Strikingly, these Rpd3‐regulated ORFs were on average marked with a relatively high level of H3K79me1 and low H3K79me3 in the wild‐type strains but became more similar to the average yeast gene upon loss of Rpd3, consistent with the presence of a negative regulator of H3K79me acting on these ORFs. Next, we compared the genes with H3K79me changes with published data on Rpd3 binding and H4 acetylation (McKnight *et al*, 2015; Data ref: McKnight *et al*, 2015). The genes with the strongest increase in H3K79me3 upon Rpd3 loss had the highest Rpd3 occupancy, both at the promoter and in the 500‐bp window downstream of the TSS (group IV; Fig 2A). Rpd3 was also found to be active at these genes, since they were devoid of H4 acetylation in wild‐type cells and H4 acetylation was restored in the *rpd3Δ* mutant (Fig 2A). The role of the deacetylase activity of Rpd3 was confirmed by ChIP‐qPCR analysis of two previously characterized mutants of Rpd3 that lack catalytic activity (Kadosh & Struhl, 1998; Sun & Hampsey, 1999). While re‐expression of wild‐type Rpd3 in the *rpd3Δ* strain restored low H3K79me3 levels at Rpd3 target genes, the Rpd3‐H188A and Rpd3‐H150A‐H151A mutants did not rescue the loss of *RPD3* (Fig 2B). Therefore, we conclude that the Rpd3 controls H3K79 methylation via its deacetylase activity. Finally, the top‐regulated genes were also enriched for meiotic genes, which are known as Rpd3 targets, and binding sites of Ume6, the Rpd3L subunit known to recruit Rpd3 to early meiotic genes (Fig 2C and D) (Kadosh & Struhl, 1998; Rundlett *et al*, 1998; Carrozza *et al*, 2005; Lardenois *et al*, 2015). Together, our results suggest that the genes at which Rpd3 restricts the buildup of H3K79me are direct targets of Rpd3.

Notably, a small subset of genes loses H3K79me3 in the absence of Rpd3 (Fig 1F, group I in Fig 2A). This group of genes already has low H3K79me3 and H2Bub levels in wild‐type cells and is highly enriched for subtelomeric genes (Fig 2A and E). Loss of Rpd3 is known to enhance Sir‐mediated silencing at subtelomeric regions (Ehrentraut *et al*, 2010, 2011; Gartenberg & Smith, 2016; Thurtle‐Schmidt *et al*, 2016). Our findings show that the stronger transcriptional silencing occurs with a concomitant reduction in H3K79me3 and H2Bub in the coding regions of heterochromatic genes. Whether or not the loss of these modifications contributes to the stronger silencing in *rpd3Δ*/*sin3Δ* mutants or is a consequence of it remains to be determined.

### Strong repression of H3K79me by Rpd3 coincides with repression of H2Bub and transcription

To understand the mechanistic basis for the crosstalk between Rpd3 and Dot1, we looked into other known functions of Rpd3 and other known regulators of H3K79me. Given the role of Rpd3 in repressing antisense transcription (Venkatesh *et al*, 2013; Castelnuovo *et al*, 2014; Murray *et al*, 2015), we compared our H3K79me data with data on antisense transcription in wild‐type cells (as calculated by Brown *et al* (2018) using data from Churchman and Weissman (2011)). This analysis showed that Rpd3 does not specifically affect H3K79 methylation at genes with high or low antisense transcription, which agrees with the notion that Rpd3 represses antisense transcription via the Rpd3S complex (Venkatesh *et al*, 2013; Castelnuovo *et al*, 2014; Murray *et al*, 2015) while it regulates H3K79me via the Rpd3L complex (Fig 1B). We also compared the changes in H3K79 methylation in mutants lacking Rpd3 with changes in H3K79 methylation in mutants lacking INO80 (Data ref: Xue *et al*, 2015). This chromatin remodeler has been shown to keep transcription and H3K79me3 at intergenic regions at bay (Xue *et al*, 2015, 2017). Our analysis shows that inactivation of the INO80 complex by deletion of *ARP5* does not specifically affect H3K79 methylation at the promoters of genes that are also regulated by Rpd3, although INO80 might affect Rpd3 target genes somewhat more than non‐target genes (Fig EV2F). This finding is in agreement with the observation that INO80 affects the majority of genes in the yeast genomes (Xue *et al*, 2015, 2017) whereas Rpd3 regulates H3K79 methylation at a smaller subset of genes. Finally, we examined the role of H2B ubiquitination in mediating the effect of Rpd3. The expression of the H2Bub machinery is not deregulated in Rpd3L mutants (Kemmeren *et al*, 2014), and no upregulation of H2Bub could be detected by immunoblot (Fig EV1A). Because subtle changes can be missed by blot, we proceeded to generate H2Bub ChIP‐seq data in wild‐type and mutant strains using an antibody we recently described (Van Welsem *et al*, 2018). We found that the strongest H3K79me3 repression by Rpd3 (group IV) coincided with repression of H2Bub as well as transcription (Fig 2A; RNA‐seq data from McKnight *et al* (2015); Data ref: McKnight *et al*, 2015). Moreover, these genes had below‐average H2Bub and transcription levels in wild‐type cells (Fig 2A). H2Bub changes were confirmed by ChIP‐qPCR (Fig EV2B and C).

The H2B ubiquitination machinery is known to be recruited co‐transcriptionally and promote H3K79 methylation, so transcriptional repression provides a likely explanation for the restriction of H3K79 methylation at these genes. However, despite these established causal links, there is no simple linear relation between transcription level and H3K79me3 level, while H2Bub correlates with transcription perfectly (Fig EV2D; Schulze *et al*, 2009, 2011; Weiner *et al*, 2015). It appears that other processes counteract H3K79 methylation (see Discussion), especially at highly transcribed genes, but that these processes do not affect the Rpd3‐regulated genes as much, since they form a subset of genes at which transcription and H3K79me3, and their changes upon *RPD3* deletion, are correlated.

In addition to genes where Rpd3 has a strong effect on H3K79me, we also observed genes at which H3K79 methylation was more modestly affected by the deletion of *RPD3* (group III; Fig 2A). Rpd3 is found at the promoters of these genes, but H4 acetylation, transcription, and H2B ubiquitination are not affected (Fig 2A). Although some of the differences may be caused by differences in antibody strength, the results suggest that at these loci, another, still unknown additional mechanism could be at play.

Taken together, we identified Rpd3 as a bona fide negative regulator of H3K79 methylation in yeast that restricts H3K79me3 at its euchromatic targets, probably mostly by repressing target gene transcription and H2Bub, but other mechanisms seem to be involved as well.

### HDAC1 loss increases H3K79me in murine thymocytes

**Figure 3:**
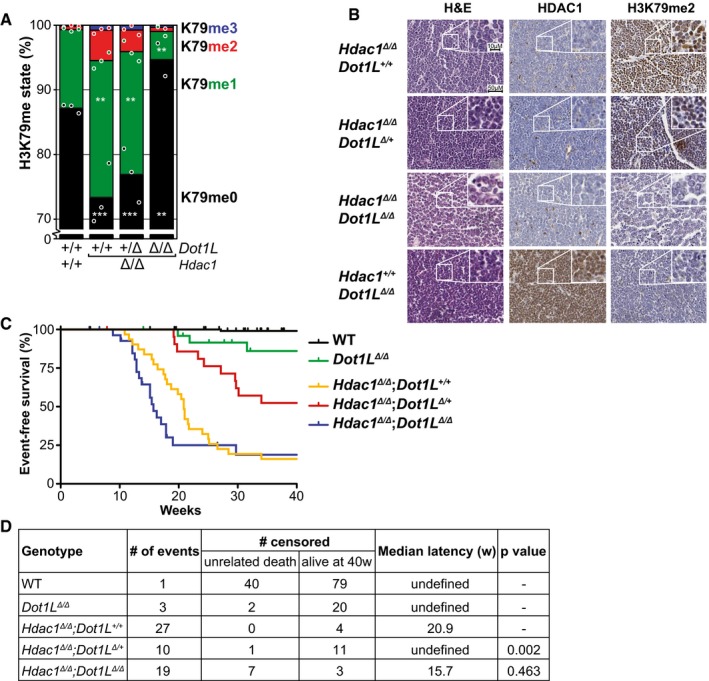
*Hdac1* deletion increases H3K79 methylation in thymocytes *in vivo*, and simultaneous heterozygous *Dot1L* deletion prolongs tumor‐free survival Mass spectrometry analysis of H3K79 methylation in thymuses from 3‐week‐old mice, either wild‐type (Cre‐) or with deleted *Hdac1* or *Dot1L* alleles, as indicated. Mean and individual data points of biological replicates; H3K79me0 is the predominant state, and the y‐axis is truncated at 70% for readability. The remaining H3K79 methylation after homozygous *Dot1L* deletion is probably due to the presence of some cells in which Cre is not expressed (yet). **P < 0.01 and ***P < 0.001 by two‐way ANOVA, comparison to wild type.Representative H&E and immunohistochemical staining on sequential sections of thymic lymphomas of the indicated genotypes. A picture with lower magnification of independent samples is included in Fig EV3B.Kaplan–Meier curves of mice harboring thymocytes with indicated genotypes. An event was defined as death or sacrifice of a mouse caused by a thymic lymphoma. Mice that died due to other causes or were still alive and event‐free at the end of the experiment were censored. Mice for which the cause of death could not be determined were removed from the data. Wild‐type mice were the Cre‐ littermates of the mice that were used for the other curves.Summary of the data presented in panel C. A median latency could only be calculated when the tumor incidence was > 50%. The *P* value was determined by comparing to the *Lck*‐Cre;*Hdac1* ^f/f^ curve with a Peto test, but a logrank test yielded the same conclusions. Source data are available online for this figure.

**Figure EV3:**
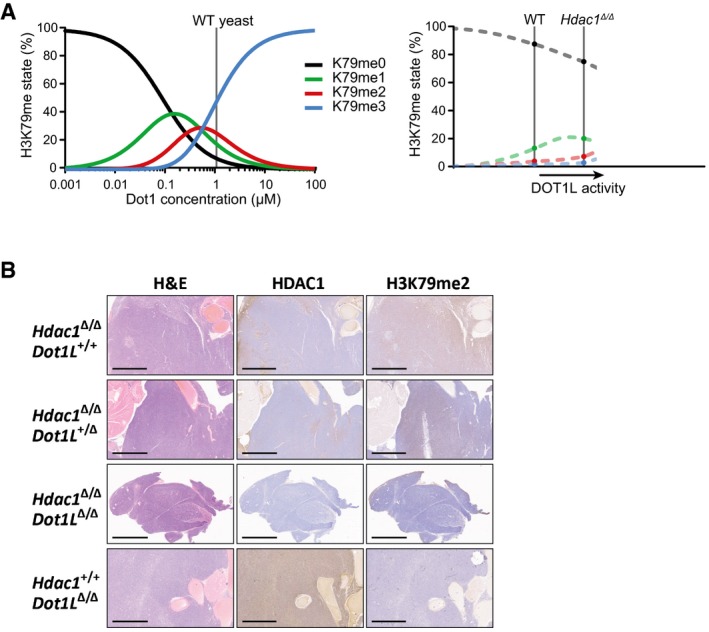
The distributive nature of Dot1/DOT1L and thymus immunohistochemistry Yeast Dot1 is a distributive enzyme and shows waves of the different methylation states over a range of Dot1 concentrations in yeast (De Vos *et al*, 2011), Fig from De Vos *et al* (2017). The catalytic nature of mammalian DOT1L enzymes is not known, but the observation that the abundance of each methylation state increases upon *Hdac1* deletion does not conflict with a distributive nature. Since H3K79 methylation levels are low in mammalian cells, it is possible we are looking at the start of the methylation waves, as indicated by the dotted lines.Representative H&E and immunohistochemical staining of sequential sections of thymic lymphomas of the indicated genotypes. The scale bar represents 2 mm.

Having uncovered a role for Rpd3 in restricting H3K79me at its targets and finding that this can explain a significant part of the H3K79 methylation variance between genes in yeast, we next wanted to investigate the biological relevance of this regulation in mammals. Histone deacetylases are conserved between species and can be divided into four classes (Yang & Seto, 2008). Rpd3 is a founding member of the class I HDACs, which in mammals includes HDAC1, HDAC2, HDAC3, and HDAC8. Of these, HDAC1 and HDAC2 are found in Sin3 complexes, like yeast Rpd3 (Yang & Seto, 2008). Given that both HDACs and DOT1L play critical roles in T‐cell malignancies, we employed conditional early thymocyte‐specific *Hdac1* deletion (*Lck*‐Cre;*Hdac1* ^f/f^, resulting in *Hdac1* ^*Δ*/*Δ*^ thymocytes) in the mouse to investigate whether the regulation that we observed in yeast also exists in T cells. We focused on HDAC1 because it is more active in mouse thymocytes than HDAC2 (Dovey *et al*, 2013; Heideman *et al*, 2013). First, we measured the relative abundance of H3K79 methylation states on bulk histones by mass spectrometry in wild‐type and *Hdac1*‐deleted thymocytes of 3‐week‐old mice (Fig 3A). In general, the overall levels of H3K79 methylation were much lower than in yeast and H3K79me1 was the most abundant methylation state, followed by H3K79me2, consistent with previous reports in mouse and human cells (Jones *et al*, 2008; Leroy *et al*, 2013). As seen in Fig 3A, *Hdac1*‐deleted thymocytes had more H3K79me2 and H3K79me1 and less H3K79me0. Considering the distributive activity of Dot1 enzymes (Fig EV3A), this suggests that Rpd3/HDAC1 is a conserved negative regulator of H3K79 methylation.

### Reduced DOT1L dosage increases the latency of *Hdac1*‐deficient thymic lymphomas

Conditional *Lck*‐Cre;*Hdac1* ^f/f^ knock‐out mice die of thymic lymphomas characterized by loss of p53 activity and *Myc* amplification, with a 75% incidence and a 23‐week mean latency (Heideman *et al*, 2013). Oncogenic transformation has not occurred yet in 3‐week‐old mice (Heideman *et al*, 2013), the age at which H3K79me levels were determined above. Since *Hdac1* deletion in thymocytes resulted in an increase in H3K79 methylation, as well as thymic lymphoma formation, we asked whether increased H3K79 methylation was important for tumor development in this mouse model. To address this question, a conditional *Dot1L* (*Dot1L* ^f/f^) allele was crossed into the *Lck*‐Cre;*Hdac1* ^f/f^ line such that deletion of *Hdac1* was combined with deletion of zero, one or two *Dot1L* alleles. Immunohistochemistry confirmed the loss of HDAC1 at the protein level, and mass spectrometry confirmed the loss of DOT1L protein activity for the expected genotypes (Figs 3A and B, and EV3B). H3K79me2 was used as an indicator for DOT1L presence, since none of the DOT1L antibodies we tested worked for IHC (antibody difficulties have also been described by Sabra *et al*, 2013).

Mice with conditional *Hdac1* alleles but wild type for *Dot1L* (*Lck*‐Cre;*Hdac1* ^f/f^) developed thymic lymphomas for which they had to be sacrificed, with a median latency of 21 weeks and an incidence of 86% during the 40‐week length of the experiment, comparable to what was observed before (Fig 3C and D) (Heideman *et al*, 2013). As expected, *Lck*‐Cre‐negative control mice rarely developed thymic lymphomas (1 out of 112). Also, *Dot1L* deletion alone (*Lck*‐Cre;*Dot1L* ^f/f^) rarely led to thymic lymphomas, with a 15% incidence in this background (Fig 3C and D), and no cases of thymic lymphoma in another background (data not shown). We then assessed the effect of *Dot1L* deletion in the *Lck*‐Cre;*Hdac1* ^f/f^ model. Loss of one copy of *Dot1L* increased survival rate and tumor latency (48% incidence, comparison to *Hdac1* ^f/f^ alone *P* = 0.002; Fig 3C and D). This effect suggests that there is a causal link between the increase in H3K79 methylation and the development or maintenance of thymic lymphomas upon *Hdac1* deletion. Interestingly, homozygous *Dot1L* deletion, leading to a complete loss of H3K79me, did not extend the latency of thymic lymphomas (81% incidence, 15.7‐week median latency, comparison to *Hdac1* deletion alone *P* = 0.463; Fig 3C and D). A possible explanation is that the simultaneous deletion of *Dot1L* and *Hdac1* results in the generation of a different class of tumor that does not depend on H3K79 methylation but has acquired other, possibly epigenetic, events that allow oncogenic transformation. A similar model has been proposed for the loss of *Hdac2* in the *Lck*‐Cre;*Hdac1* ^f/f^ model (Heideman *et al*, 2013). For the heterozygous *Dot1L* effect, we consider two possible explanations: The oncogenic transformation occurred later because an additional event was required to overcome the lack of high H3K79me, or tumors grew slower due to lower H3K79me, because of either decreased proliferation or increased apoptosis.

### *Hdac1*‐deficient thymic lymphoma lines depend on DOT1L activity

**Figure 4:**
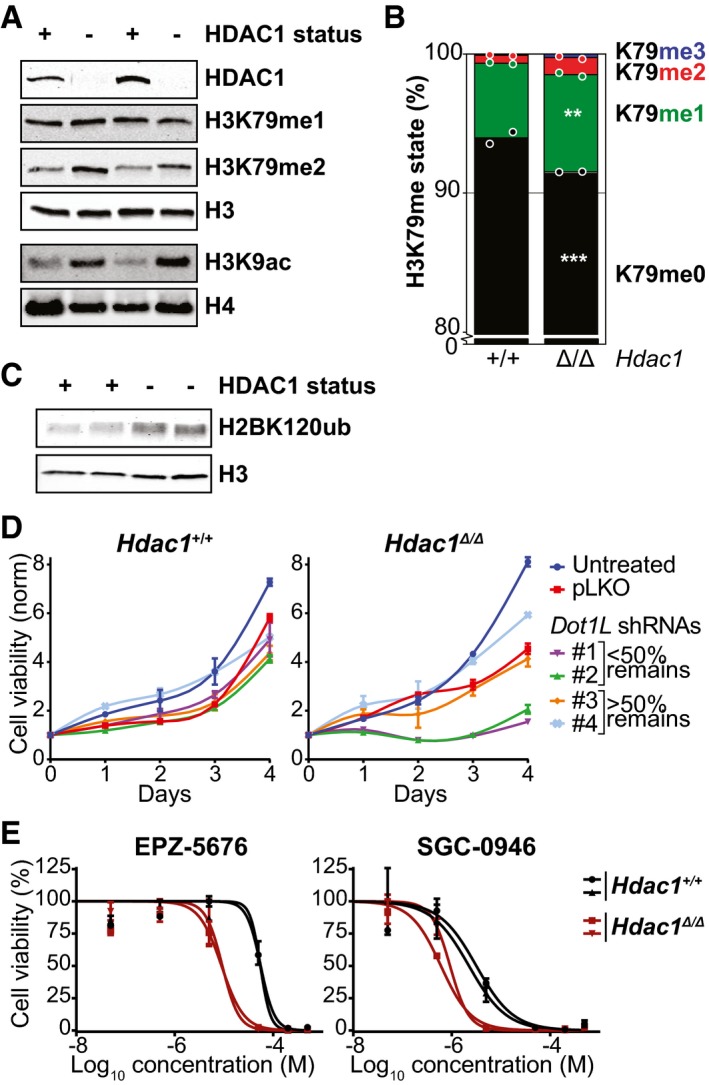
*Hdac1*‐deleted thymic lymphoma cell lines depend on DOT1L activity Immunoblots showing HDAC1 status and H3K79me/H3K9ac levels in nuclear lysates of *Hdac1*‐proficient (p53‐null) and *Hdac1*‐deficient thymic lymphoma cell lines. The top four and bottom two panels are from separate lysates of the same cell lines.Mass spectrometry analysis of H3K79 methylation in the cell lines from panel A. Mean and individual data points of two independent cell lines; H3K79me0 is the predominant state, and the y‐axis is truncated at 80% for readability. **P < 0.01 and ***P < 0.001 by two‐way ANOVA.Immunoblot showing H2BK120 ubiquitination levels in Hdac1‐proficient and Hdac1‐deficient cell lines (two independent lines each).Growth curves of *Hdac1*‐proficient and *Hdac1*‐deficient cell lines that were left untreated or were infected with empty virus (pLKO) or shRNAs against *Dot1L* and selected with puromycin. Growth curves were determined by a series of resazurin assays, which measure metabolic activity, starting from four days post‐infection. Error bars indicate the range of two replicates from independent cell lines.Inhibitor dose–response curves of the two DOT1L inhibitors EPZ‐5676 (Pinometostat) and SGC‐0946 in *Hdac1*‐proficient and *Hdac1*‐deficient cell lines. Cell viability was measured by a resazurin assay after three days of treatment, and measurements were normalized to DMSO‐treated cells. Two independent cell lines are plotted separately; error bars indicate the range of two biological replicates. Source data are available online for this figure.

**Figure EV4:**
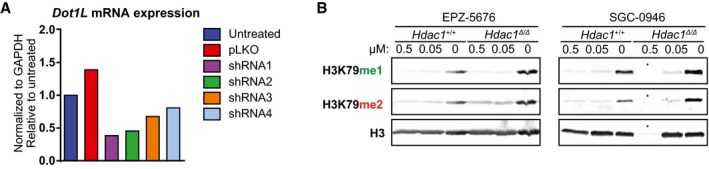
Control experiments belonging to Fig 4 Knockdown efficiency of the shRNAs used in Fig 4D. The shRNAs 1 and 2 give a knockdown of more than 50%.Immunoblot analysis of H3K79 methylation levels in the two cell lines with and without inhibitors. Both the effect of the inhibitors and the difference between the cell lines can be observed; * indicates a lane that was underloaded because most cells had died. For cells with EPZ‐5676 treatment, the H3K79me1 blot was re‐probed with α‐H3K79me2, following stripping the previous antibody and confirming that the stripping was complete. Finally, the blot was re‐probed with α‐H3‐C. Source data are available online for this figure.

To further study the *Dot1L* dependence of *Hdac1*‐deficient thymic lymphomas in a more controlled environment, we turned to *ex vivo* experiments. Cell lines were derived from *Hdac1*‐deficient thymic lymphomas (Heideman *et al*, 2013). Since these cell lines had an inactivating mutation in p53, cell lines derived from p53‐null thymic lymphomas were used as *Hdac1*‐proficient control lines (Heideman *et al*, 2013). First, we examined whether *Hdac1*‐deficient tumor cells retained the increased H3K79 methylation levels seen prior to the oncogenic transformation. Both by immunoblot and by targeted mass spectrometry on independent samples (Fig 4A and B), *Hdac1*‐deficient tumor cell lines had more H3K79 methylation than their *Hdac1*‐proficient counterparts. Thus, the effect of HDAC1 on H3K79 methylation observed *in vivo* in 3‐week‐old pre‐malignant thymuses was maintained in the thymic lymphoma cell lines. Importantly, *Hdac1*‐deficient cell lines also possessed high levels of ubiquitinated H2B compared to *Hdac1*‐proficient controls (Fig 4C). High H2Bub is consistent with the increase in H2Bub seen at Rpd3 targets in yeast. Therefore, a plausible model is that at least part of the observed increase in H3K79me upon loss of HDAC1 activity is mediated via H2Bub, consistent with what we observed in yeast. However, contributions from other regulatory mechanisms cannot be excluded (see Discussion). To test the DOT1L dependence of the cell lines, DOT1L was depleted using shRNAs in *Hdac1*‐proficient and *Hdac1*‐deficient cell lines. As can be seen in Fig 4D, shRNAs that reduce Dot1L expression (Fig EV4A) affected proliferation of the *Hdac1*‐deficient cell lines. Compared to the control lines, the *Hdac1*‐deficient cell lines were also more sensitive to two different DOT1L inhibitors (Fig 4E). Both inhibitors, EPZ‐5676 (Pinometostat) and SGC‐0946, effectively lowered H3K79 methylation (Fig EV4B). Thus, shRNA‐mediated DOT1L knockdown and chemical DOT1L inhibition showed that the *Hdac1*‐deficient thymic lymphoma cell lines depended on DOT1L activity.

**Figure 5:**
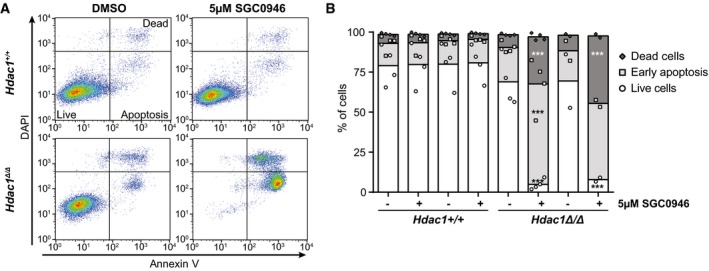
*Hdac1*‐deficient thymic lymphoma lines require DOT1L activity for survival Representative apoptosis FACS plots of cell lines treated with DMSO or the DOT1L inhibitor SGC‐0946 for two days. Annexin V staining and DAPI staining were performed on unpermeabilized cells to distinguish live (Annexin V low; DAPI low), apoptotic (Annexin V high; DAPI low), and dead (Annexin V high; DAPI high) cells.Quantification of several independent FACS experiments, including the experiment shown in panel (A). Mean with individual data points of 2–4 replicates each of two independent lines per genotype. ***P < 0.001 by two‐way ANOVA, comparison to corresponding DMSO control. Source data are available online for this figure.

The reduced growth upon inactivation of DOT1L could be explained by a block in proliferation or an increase in cell death. To measure apoptosis induction, the levels of Annexin V and DAPI staining of non‐permeabilized cells were determined by flow cytometry. In the DMSO‐treated condition, most cells were alive, although *Hdac1*‐deficient lines had a slightly higher basal apoptosis level (Fig 5A and B). This combination of proliferation and apoptosis has also been observed in *Hdac1* ^Δ/Δ^ teratomas (Lagger *et al*, 2010). However, DOT1L inhibition by 5μM of SGC‐0946 dramatically induced apoptosis in *Hdac1*‐deficient cells, whereas no effect on apoptosis was observed in the control cell lines (Fig 5A and B). Thus, DOT1L inhibition induced apoptosis specifically in *Hdac1*‐deficient thymic lymphoma cell lines.

## Discussion

Here, we describe that the yeast HDAC, Rpd3, is a negative regulator of H3K79 methylation that restricts methylation at the 5′ ends of its target genes. Similar to what we observe for Rpd3 in yeast, deleting *Hdac1* in murine thymocytes leads to an increase in H3K79 methylation. This regulation is relevant in a cancer context, since heterozygous deletion of *Dot1L* prolongs the survival of mice that develop *Hdac1*‐deficient thymic lymphomas. Cell lines derived from *Hdac1*‐deficient thymic lymphomas undergo apoptosis upon DOT1L inhibition or depletion, which indicates a form of non‐oncogene addiction to DOT1L.

### Rpd3 target genes

In the yeast genome, most euchromatic genes are marked by H2Bub and H3K79me3 in their transcribed region. While the levels of H2Bub correlate well with transcription levels, it is evident that H2Bub is not the only determinant of H3K79me3 in yeast because the relation between transcription and H3K79me3 is more complex (Fig 2A). While genes silenced by the SIR complex have low H3K79me3 levels due to active repression mechanisms (Gartenberg & Smith, 2016), the majority of euchromatic genes contain H3K79me3 irrespective of their expression level (Fig EV2D; Schulze *et al*, 2009, 2011; Weiner *et al*, 2015). Some genes contain lower H3K79me3 and higher H3K79me1 levels than the average gene, however. A subset of these deviants has been identified as genes undergoing antisense transcription (Murray *et al*, 2015; Brown *et al*, 2018), possibly resulting in nucleosome instability and increased histone turnover, which counteracts the buildup of higher H3K79me states but does not affect the more dynamic H2Bub modification (Weiner *et al*, 2015). Here, we provide insight into the low H3K79me3/high H3K79me1 levels of another subset of yeast genes. H3K79me ChIP‐seq in yeast revealed that Rpd3 restricts H3K79me3 at its direct target genes. This subset of genes showed great overlap with the minority of euchromatic genes that is marked with H3K79me1 instead of H3K79me3. Thus, the regulation by Rpd3 provides an explanation for the variation in H3K79 methylation between genes and thereby seems to be an important determinant of the H3K79 methylation pattern. The H3K79me effect of Rpd3 was most notable at the 5′ end of genes, which is in agreement with previous studies on Rpd3 activity. The deacetylation activity of Rpd3/Rpd3L is reported to be strongest in coding sequences, particularly at the 5′ ends (Weinberger *et al*, 2012). At which genes HDAC1 regulates H3K79 methylation in murine thymocytes is an interesting question, but addressing it is not straightforward. Unlike in yeast, in mammals H3K79 methylation is tightly linked to the transcriptional activity at genes. Processes through which transcription promotes H3K79 methylation are known, but in turn, H3K79 methylation may affect transcription as well (reviewed in Vlaming & Van Leeuwen, 2016). Therefore, when assessing H3K79me changes in *Hdac1*‐deficient cells, it will be challenging to separate direct effects from indirect effects on DOT1L activity through transcriptional changes.

### Mechanism of regulation

What could be the mechanism of H3K79me regulation by Rpd3/HDAC1? Until now, the H2B ubiquitination machinery was the only described H3K79me regulator conserved from yeast to mammals (Weake & Workman, 2008). Here, we describe another conserved regulator, Rpd3/HDAC1, and our results indicate that it acts in part by restricting H2Bub. Although transcription and H3K79 methylation are not clearly positively correlated in wild‐type yeast cells (Fig EV2D; Schulze *et al*, 2011; Magraner‐Pardo *et al*, 2014; Weiner *et al*, 2015), we observed that the H3K79 methylation changes in *rpd3Δ* correlate very well with transcriptional changes. These data, together with the well‐established causal relationships between transcription and H2B ubiquitination on the one hand and H2B ubiquitination and H3K79 methylation on the other hand, suggest that there is indeed a causal link between (sense) transcription and the placement of H3K79 methylation at a subset of the yeast genome. At higher transcription levels however, this relationship can be obscured by other processes, most likely histone turnover, counteracting the high Dot1 activity (Radman‐Livaja *et al*, 2011; Murray *et al*, 2015). We have recently identified the conserved histone acetyltransferase Gcn5 as a negative regulator of H3K79me and H2Bub (Vlaming *et al*, 2016). At first glance, it seems counterintuitive that a HAT and an HDAC have overlapping effects. However, acetylation at non‐overlapping histone or non‐histone lysines may explain this discrepancy. For example, Gcn5 most likely negatively regulates H2Bub and H3K79me by affecting the deubiquitinating module of the SAGA co‐activator complex in which Gcn5 also resides (Vlaming *et al*, 2016). H2B ubiquitination by human RNF20/40 has also been shown to be regulated by histone acetylation (Garrido Castro *et al*, 2018). Treatment of acute lymphoblastic leukemia cell lines with the non‐selective HDAC inhibitor Panobinostat showed changes in H2Bub, with decreased H2Bub in MLL‐r leukemia lines and increased H2Bub in non‐MLL‐r leukemia lines, suggesting context‐dependent mechanisms (Garrido Castro *et al*, 2018).

Besides H2Bub, other mechanisms are likely to contribute to the observed H3K79me increase in the absence of Rpd3/HDAC1 as well. Histone acetylation is increased in the absence of the deacetylase HDAC1, and histone acetylation has been previously linked to DOT1L recruitment through the YEATS domain transcription elongation proteins AF9 and ENL (Li *et al*, 2014; Kuntimaddi *et al*, 2015; Erb *et al*, 2017; Wan *et al*, 2017). Very recently, preferential Dot1 binding to acetylated H4K16 has been shown, and the histone acetyltransferase Sas2 was found to be a positive regulator of H3K79 methylation in yeast, probably via acetylation of H4K16 (Lee *et al*, 2018). Our identification of Rpd3/HDAC1 as a regulator of DOT1L underscores the intimate relationship between histone acetylation and H2Bub and H3K79me and provides evidence for a specific HDAC involved in the crosstalk: HDAC1.

### DOT1L in tumor maintenance

Loss of HDAC1 leads to oncogenic transformation and higher H3K79me in murine thymocytes. Heterozygous *Dot1L* deletion prolonged the survival of mice with thymocyte‐specific *Hdac1* deletion due to a lower incidence and increased latency of thymic lymphomas. Our analysis of *Hdac1*‐deficient thymic lymphoma cells *ex vivo* provided more insight into the possible mechanisms for the reduced tumor burden. Using DOT1L inhibitors and a knockdown approach, we established that DOT1L was required for survival of the tumor cells by preventing the induction of apoptosis, suggesting that DOT1L is required for tumor maintenance. The DOT1L dependency of the thymic lymphomas resembles that of MLL‐rearranged leukemia (Wang *et al*, 2016) as well as breast and lung cancer cell lines (Kim *et al*, 2012; Zhang *et al*, 2014). The full genetic deletion of *Dot1L* did not reduce tumor burden. The reasons for this are currently unknown and require further study. One possible reason is that some remaining DOT1L activity and H3K79 methylation might be required to induce apoptosis in the tumor cells. We note that in the *ex vivo* experiments where *Dot1L* knockdown and DOT1L inhibitors were found to lead to induction of apoptosis; some residual H3K79me was indeed still present. Another possibility is that the simultaneous loss of *Hdac1* and *Dot1L* imposes oncogenic transformation through alternative, epigenetic mechanisms that bypass the apoptotic‐prone state. This would be in agreement with the known role of DOT1L in the maintenance of cellular epigenetic states (Onder *et al*, 2012; Soria‐Valles *et al*, 2015; Breindel *et al*, 2017). Regardless of possible mechanisms, the finding that HDAC1 affects DOT1L activity in yeast as well as mouse T cells warrants further investigation. For example, it will be interesting to determine whether and under which conditions HDAC1 activity influences DOT1L activity in human MLL‐r leukemia and whether the crosstalk is involved in the response of CTLC to HDAC inhibitors in the clinic. Our findings in murine lymphoma add to a growing list of cancers that rely on DOT1L activity, and therefore underline the importance of understanding the regulation of DOT1L.

## Materials and Methods

### Yeast strains and plasmids

Yeast strains used in this article are listed in Appendix Table S1. Yeast media were described previously (Van Leeuwen & Gottschling, 2002). The generation of the barcoded deletion library used for the Epi‐ID experiment was described previously (Vlaming *et al*, 2016). Yeast *rpd3Δ* and *sin3Δ* strains were taken from this library and independent clones were generated by deleting these genes in the barcoded wild‐type strain NKI4657, using the NatMX selection marker from pFvL99 (Stulemeijer *et al*, 2011). Gene deletions were confirmed by PCR. *RPD3* expression vectors were derived from YCplac22‐RPD3, YCplac22‐rpd3_H188A, and YCplac112‐rpd3_H150A_H151A (Kadosh & Struhl, 1998). The inserts were released by digestion with *Sac*I and *Sal*I and cloned into the same sites of the single‐copy *LEU2* vector pRS315 (Brachmann *et al*, 1998). The mutations were verified by DNA sequencing. Strains harboring pRS315‐derived plasmids were grown in synthetic media lacking leucine.

### Cell culture

Thymic lymphoma cell lines (NKI8996, NKI9002) were derived from *Hdac1*‐deficient thymic lymphomas (Heideman *et al*, 2013). Since these cell lines had an inactivating mutation in p53, cell lines derived from p53‐null thymic lymphomas (NKI8995, NKI8999) were used as *Hdac1*‐proficient control lines (Heideman *et al*, 2013). Thymic lymphoma cell lines were cultured under standard conditions in RPMI 1640 (Gibco) supplemented with 10% FBS (Sigma‐Aldrich), antibiotics, and L‐glutamine. The HDAC1 status was confirmed by immunoblot analyses (Fig 4A). HEK 293T cells were cultured in DMEM (Gibco) supplemented with 10% FBS (Sigma‐Aldrich) and l‐glutamine. Cell lines were regularly tested for mycoplasma contamination.

### Mouse survival analysis

The generation and crosses of the conditional knock‐out mice are described in the Appendix Supplementary Methods. Mice were monitored over time, up to 40 weeks of age. A power analysis performed beforehand determined group sizes of 20–25 mice per genotype. Mice were sacrificed before 40 weeks when they displayed breathing issues caused by the thymic lymphoma, or serious discomfort unrelated to tumor formation. After death or sacrifice, mice were checked for the presence of a thymic lymphoma. Mice that died without a lymphoma were censored in the survival curves. Mice of which the cause of death could not be determined were left out of the survival curve, together with their littermates. Mice were kept in individually ventilated cages at the animal laboratory facility of the Netherlands Cancer Institute (NKI; Amsterdam, the Netherlands). Food and water were provided ad libitum. Animal experiments were approved by the Animal Ethics Committee of the NKI and performed in accordance with institutional, national, and European guidelines for animal care and use. The study is compliant with all relevant ethical regulations regarding animal research.

### Protein lysates, immunoblots, and mass spectrometry

Yeast whole‐cell extracts were made as described previously (Vlaming *et al*, 2014). Nuclear extracts were prepared of murine cells; see Appendix Supplementary Methods for details. The immunoblotting procedure was as described in Vlaming *et al* (2014). All antibodies used in this study are listed in the Appendix Supplementary Methods. Mass spectrometry measurements on yeast strains and thymic lymphoma cell lines were as described in Vlaming *et al* (2014). Measurements on thymus tissue were performed using the method described in Vlaming *et al* (2016).

### ChIP‐sequencing and ChIP‐qPCR

ChIP and ChIP‐qPCR experiments were performed as described before (Vlaming *et al*, 2016). Primers for qPCR are shown in Appendix Table S2. Details on the ChIP‐seq library preparation and the first analysis steps are provided in the Appendix Supplementary Methods. In short, preparation and sequencing were performed by the NKI Genomics Core Facility. Reads were mapped to the *Saccharomyces cerevisiae* reference genome R64‐2‐1 and extended to 150 bp. Samples were depth‐normalized, and when data from biological duplicates were found to be similar, data sets were merged for further analyses. Metagene plots and heatmaps were generated with custom scripts in R/Bioconductor (Cherry *et al*, 2012; Huber *et al*, 2015). Reads were aligned in a window of −500 to 1 kb around the TSS of each verified ORF recorded in SGD. Genes that contained a coverage of 0 or an average coverage in the first 500 bp below 0.5 were filtered out (leaving 5,006 out of 5,134 genes). For heatmaps, the coverage was grouped in bins of 10 bp. The plots in Figs 2A and EV2D–F were created with custom scripts in R by using locally weighted regression (LOESS). Transcription in WT cells was obtained from McKnight *et al* (2015) and transcription in *rpd3Δ*/WT from Kemmeren *et al* (2014). Gene set enrichment plots were created with the barcodeplot function from the limma package (Ritchie *et al*, 2015). Ume6 targets filtered for Ume6 DNA binding were obtained from the YEASTRACT database (Teixeira *et al*, 2014). The list of meiosis factors was generated by searching for genes with a GO term containing “meio” or children thereof. The distance of each gene from the telomere on the same chromosome arm was calculated manually by using genome feature information from SGD.

### Histology/immunohistochemistry

Tissues were fixed in EAF (ethanol, acetic acid, and formol saline) for 24 h and subsequently embedded in paraffin. Slides were stained with hematoxylin and eosin (H&E), or immunohistochemistry was performed as described (Heideman *et al*, 2013).

### Knockdown/viability assays and Dot1L mRNA analysis

To produce lentiviral particles, HEK 293T cells were co‐transfected with shRNA‐containing pLKO.1 and three packaging plasmids containing Gag and Pol, and Rev and VSV‐G, respectively, using PEI. The media were replaced after 16 h, and virus‐containing medium was harvested 72 h after transfection. Virus particles were 10× concentrated from filtered medium using Amicon 100 kDa spin columns. For lentiviral infections, 100,000 cells were seeded in 96‐well tissue culture plates and infected using 7.5 μl concentrated virus, in the presence of 8 μg/ml polybrene. The medium of infected cells was replaced with puromycin‐containing medium 48 h after infection and refreshed again 72 h after infection, after which a cell viability assay was performed every 24 h. Cell viability was determined by a CellTiter‐Blue (Promega) assay, measuring conversion to resorufin after 3 h with the EnVision Multilabel Reader (PerkinElmer). All cells treated with a particular shRNA were pooled for RNA isolation using the RNeasy Mini Kit (Qiagen). DNase I (New England Biolabs) digestion was performed, and RNA was reverse‐transcribed into cDNA using SuperScript II Reverse Transcriptase (Invitrogen) according to the manufacturer's protocol. *Dot1L* transcript abundance was measured by qPCR using SYBR Green Master Mix (Roche) and the LightCycler 480 II (Roche). Primers for qPCR can be found in Appendix Table S2.

### Inhibitor treatment

A total of 100,000 cells were seeded in 96‐well tissue culture plates, in 200 μl culture medium containing the indicated concentration of inhibitor. Two inhibitors were used: SGC‐0946 (Structural Genomics Consortium) and EPZ‐5676 (Pinometostat; Selleck Chemicals). Cell viability was determined after three days, as described above. Data were normalized, with the maximum of each cell line to 100% and background fluorescence set to 0%. GraphPad Prism was used to fit log(inhibitor) vs normalized response curves with a variable slope.

### Apoptosis FACS

Cells treated with 0.1% DMSO or 5 μM SGC‐0946 were stained with Annexin V‐FITC and DAPI following the protocol of the Annexin V‐FITC Apoptosis Detection Kit (Abcam). Fluorescence was detected by FACS using the CyAn ADP Analyzer (Beckman Coulter), and data were analyzed using FlowJo software.

### Statistics

Survival curves were plotted in GraphPad Prism, and Peto mortality‐prevalence tests were performed in SAS to compare the curve of *Lck*‐Cre;*Hdac1* ^f/f^ mice with the *Lck*‐Cre;*Hdac1* ^f/f^;*Dot1L* ^f/WT^ and *Lck*‐Cre;*Hdac1* ^f/f^;*Dot1L* ^f/f^ curves. The same conclusions could be drawn based on the standard logrank test in GraphPad Prism. Mass spectrometry and ChIP‐qPCR data were compared using two‐way ANOVA, comparing samples of all genotypes to the wild‐type sample and using the Dunnett's correction for multiple comparisons, using GraphPad Prism.

## Author contributions

HV, CMM, and FvL designed the studies and the cell line and mouse studies together with HJ and J‐HD. Yeast experiments were performed by HV, TMM, and TvW; mouse experiments by CMM, HV, SH, EMK‐M, MFA, and CL; and cell line experiments by CMM, SP, and HV. TK performed ChIP‐seq and genomics analyses, SK made IHC pictures and gave histology advice, and LH and TTS performed mass spectrometry measurements and were advised by AFMA. HV and FvL wrote the manuscript, with help from CMM, HJ, and J‐HD.

## Conflict of interest

The Netherlands Cancer Institute and FvL are entitled to royalties that may result from licensing the yeast H2BK123ub‐specific monoclonal antibody according to IP policies of the Netherlands Cancer Institute. The other authors declare that they have no conflict of interest.

## Supporting information



Appendix

Expanded View Figures PDF

Source Data for Expanded View

Review Process File

Source Data for Figure 1

Source Data for Figure 2

Source Data for Figure 3

Source Data for Figure 4

Source Data for Figure 5

## Data Availability

The ChIP‐seq data from this publication have been deposited to the GEO database (www.ncbi.nlm.nih.gov/geo/) and assigned the identifier accession number GSE107331.

## References

[embj2019101564-bib-0001] Bernt KM , Zhu N , Sinha AU , Vempati S , Faber J , Krivtsov AV , Feng Z , Punt N , Daigle A , Bullinger L *et al* (2011) MLL‐rearranged leukemia is dependent on aberrant H3K79 methylation by DOT1L. Cancer Cell 20: 66–78 21741597 10.1016/j.ccr.2011.06.010 PMC3329803

[embj2019101564-bib-0002] Brachmann CB , Davies A , Cost GJ , Caputo E , Li J , Hieter P , Boeke JD (1998) Designer deletion strains derived from *Saccharomyces cerevisiae* S288C: a useful set of strains and plasmids for PCR‐mediated gene disruption and other applications. Yeast 14: 115–132 9483801 10.1002/(SICI)1097-0061(19980130)14:2<115::AID-YEA204>3.0.CO;2-2

[embj2019101564-bib-0003] Breindel JL , Skibinski A , Sedic M , Wronski‐Campos A , Zhou W , Keller PJ , Mills J , Bradner J , Onder T , Kuperwasser C (2017) Epigenetic reprogramming of lineage‐committed human mammary epithelial cells requires DNMT3A and loss of DOT1L. Stem Cell Reports 9: 943–955 28781076 10.1016/j.stemcr.2017.06.019 PMC5599181

[embj2019101564-bib-0004] Brien GL , Valerio DG , Armstrong SA (2016) Exploiting the epigenome to control cancer‐promoting gene‐expression programs. Cancer Cell 29: 464–476 27070701 10.1016/j.ccell.2016.03.007 PMC4889129

[embj2019101564-bib-0005] Brocks D , Schmidt CR , Daskalakis M , Jang HS , Shah NM , Li D , Li J , Zhang B , Hou Y , Laudato S *et al* (2017) DNMT and HDAC inhibitors induce cryptic transcription start sites encoded in long terminal repeats. Nat Genet 49: 1052–1060 28604729 10.1038/ng.3889 PMC6005702

[embj2019101564-bib-0006] Brown T , Howe FS , Murray SC , Wouters M , Lorenz P , Seward E , Rata S , Angel A , Mellor J (2018) Antisense transcription‐dependent chromatin signature modulates sense transcript dynamics. Mol Syst Biol 14: e8007 29440389 10.15252/msb.20178007 PMC5810148

[embj2019101564-bib-0007] Campbell CT , Haladyna JN , Drubin DA , Thomson TM , Maria MJ , Yamauchi T , Waters NJ , Olhava EJ , Pollock RM , Smith JJ *et al* (2017) Mechanisms of pinometostat (EPZ‐5676) treatment‐emergent resistance in MLL ‐rearranged leukemia. Mol Cancer Ther 16: 1669–1679 28428443 10.1158/1535-7163.MCT-16-0693

[embj2019101564-bib-0008] Carrozza MJ , Li B , Florens L , Suganuma T , Swanson SK , Lee KK , Shia W‐J , Anderson S , Yates J , Washburn MP *et al* (2005) Histone H3 methylation by Set2 directs deacetylation of coding regions by Rpd3S to suppress spurious intragenic transcription. Cell 123: 581–592 16286007 10.1016/j.cell.2005.10.023

[embj2019101564-bib-0009] Castelnuovo M , Zaugg JB , Guffanti E , Maffioletti A , Camblong J , Xu Z , Clauder‐Münster S , Steinmetz LM , Luscombe NM , Stutz F (2014) Role of histone modifications and early termination in pervasive transcription and antisense‐mediated gene silencing in yeast. Nucleic Acids Res 42: 4348–4362 24497191 10.1093/nar/gku100 PMC3985671

[embj2019101564-bib-0010] Chen S , Yang Z , Wilkinson AW , Deshpande AJ , Sidoli S , Krajewski K , Strahl BD , Garcia BA , Armstrong SA , Patel DJ *et al* (2015) The PZP domain of AF10 senses unmodified H3K27 to regulate DOT1L‐mediated methylation of H3K79. Mol Cell 60: 319–327 26439302 10.1016/j.molcel.2015.08.019 PMC4609290

[embj2019101564-bib-0011] Cherry JM , Hong EL , Amundsen C , Balakrishnan R , Binkley G , Chan ET , Christie KR , Costanzo MC , Dwight SS , Engel SR *et al* (2012) Saccharomyces Genome Database: the genomics resource of budding yeast. Nucleic Acids Res 40: D700–D705 22110037 10.1093/nar/gkr1029 PMC3245034

[embj2019101564-bib-0012] Churchman LS , Weissman JS (2011) Nascent transcript sequencing visualizes transcription at nucleotide resolution. Nature 469: 368–373 21248844 10.1038/nature09652 PMC3880149

[embj2019101564-bib-0013] Daigle SR , Olhava EJ , Therkelsen CA , Basavapathruni A , Jin L , Boriack‐Sjodin PA , Allain CJ , Klaus CR , Raimondi A , Scott MP *et al* (2013) Potent inhibition of DOT1L as treatment of MLL‐fusion leukemia. Blood 122: 1017–1025 23801631 10.1182/blood-2013-04-497644 PMC3739029

[embj2019101564-bib-0014] De Vos D , Frederiks F , Terweij M , van Welsem T , Verzijlbergen KF , Iachina E , de Graaf EL , Altelaar AFM , Oudgenoeg G , Heck AJR *et al* (2011) Progressive methylation of ageing histones by Dot1 functions as a timer. EMBO Rep 12: 956–962 21760613 10.1038/embor.2011.131 PMC3166457

[embj2019101564-bib-0015] De Vos D , Vlaming H , Bakker BM , Van Leeuwen F (2017) Chapter 6 ‐ Modeling Distributive Histone Modification by Dot1 Methyltransferases: From Mechanism to Biological Insights. In Epigenetics and Systems Biology, Ringrose L (ed), pp 117‐141. Boston: Academic Press

[embj2019101564-bib-0016] Deshpande AJ , Deshpande A , Sinha AU , Chen L , Chang J , Cihan A , Fazio M , Chen C , Zhu N , Koche R *et al* (2014) AF10 regulates progressive H3K79 methylation and HOX gene expression in diverse AML subtypes. Cancer Cell 26: 896–908 25464900 10.1016/j.ccell.2014.10.009 PMC4291116

[embj2019101564-bib-0017] Dovey OM , Foster CT , Conte N , Edwards SA , Edwards JM , Singh R , Vassiliou G , Bradley A , Cowley SM (2013) Histone deacetylase 1 and 2 are essential for normal T‐cell development and genomic stability in mice. Blood 121: 1335–1344 23287868 10.1182/blood-2012-07-441949 PMC3836254

[embj2019101564-bib-0018] Drouin S , Laramée L , Jacques P‐É , Forest A , Bergeron M , Robert F (2010) DSIF and RNA polymerase II CTD phosphorylation coordinate the recruitment of Rpd3S to actively transcribed genes. PLoS Genet 6: e1001173 21060864 10.1371/journal.pgen.1001173 PMC2965751

[embj2019101564-bib-0019] Ehrentraut S , Weber JM , Dybowski JN , Hoffmann D , Ehrenhofer‐Murray AE (2010) Rpd3‐dependent boundary formation at telomeres by removal of Sir2 substrate. Proc Natl Acad Sci USA 107: 5522–5527 20133733 10.1073/pnas.0909169107 PMC2851772

[embj2019101564-bib-0020] Ehrentraut S , Hassler M , Oppikofer M , Kueng S , Weber JM , Mueller JW , Gasser SM , Ladurner AG , Ehrenhofer‐Murray AE (2011) Structural basis for the role of the Sir3 AAA+ domain in silencing: interaction with Sir4 and unmethylated histone H3K79. Genes Dev 25: 1835–1846 21896656 10.1101/gad.17175111 PMC3175719

[embj2019101564-bib-0021] Emre NCT , Ingvarsdottir K , Wyce A , Wood A , Krogan NJ , Henry KW , Li K , Marmorstein R , Greenblatt JF , Shilatifard A *et al* (2005) Maintenance of low histone ubiquitylation by Ubp10 correlates with telomere‐proximal Sir2 association and gene silencing. Mol Cell 17: 585–594 15721261 10.1016/j.molcel.2005.01.007

[embj2019101564-bib-0022] Erb MA , Scott TG , Li BE , Xie H , Paulk J , Seo H‐S , Souza A , Roberts JM , Dastjerdi S , Buckley DL *et al* (2017) Transcription control by the ENL YEATS domain in acute leukaemia. Nature 543: 270–274 28241139 10.1038/nature21688 PMC5497220

[embj2019101564-bib-0023] Frederiks F , Tzouros M , Oudgenoeg G , van Welsem T , Fornerod M , Krijgsveld J , van Leeuwen F (2008) Nonprocessive methylation by Dot1 leads to functional redundancy of histone H3K79 methylation states. Nat Struct Mol Biol 15: 550–557 18511943 10.1038/nsmb.1432

[embj2019101564-bib-0024] Gardner RG , Nelson ZW , Gottschling DE (2005) Ubp10/Dot4p regulates the persistence of ubiquitinated histone H2B: distinct roles in telomeric silencing and general chromatin. Mol Cell Biol 25: 6123–6139 15988024 10.1128/MCB.25.14.6123-6139.2005 PMC1168808

[embj2019101564-bib-0025] Garrido Castro P , van Roon EHJ , Pinhanços SS , Trentin L , Schneider P , Kerstjens M , te Kronnie G , Heidenreich O , Pieters R , Stam RW (2018) The HDAC inhibitor panobinostat (LBH589) exerts in vivo anti‐leukaemic activity against MLL‐rearranged acute lymphoblastic leukaemia and involves the RNF20/RNF40/WAC‐H2B ubiquitination axis. Leukemia 32: 323–331 28690313 10.1038/leu.2017.216

[embj2019101564-bib-0026] Gartenberg MR , Smith JS (2016) The nuts and bolts of transcriptionally silent chromatin in *Saccharomyces cerevisiae* . Genetics 203: 1563–1599 27516616 10.1534/genetics.112.145243 PMC4981263

[embj2019101564-bib-0027] Heideman MR , Wilting RH , Yanover E , Velds A , de Jong J , Kerkhoven RM , Jacobs H , Wessels LF , Dannenberg J‐H (2013) Dosage‐dependent tumor suppression by histone deacetylases 1 and 2 through regulation of c‐Myc collaborating genes and p53 function. Blood 121: 2038–2050 23327920 10.1182/blood-2012-08-450916 PMC3596963

[embj2019101564-bib-0028] Huber W , Carey VJ , Gentleman R , Anders S , Carlson M , Carvalho BS , Bravo HC , Davis S , Gatto L , Girke T *et al* (2015) Orchestrating high‐throughput genomic analysis with Bioconductor. Nat Methods 12: 115–121 25633503 10.1038/nmeth.3252 PMC4509590

[embj2019101564-bib-0029] Imbeault D , Gamar L , Rufiange A , Paquet E , Nourani A (2008) The Rtt106 histone chaperone is functionally linked to transcription elongation and is involved in the regulation of spurious transcription from cryptic promoters in yeast. J Biol Chem 283: 27350–27354 18708354 10.1074/jbc.C800147200

[embj2019101564-bib-0030] Jones B , Su H , Bhat A , Lei H , Bajko J , Hevi S , Baltus GA , Kadam S , Zhai H , Valdez R *et al* (2008) The histone H3K79 methyltransferase Dot1L is essential for mammalian development and heterochromatin structure. PLoS Genet 4: e1000190 18787701 10.1371/journal.pgen.1000190 PMC2527135

[embj2019101564-bib-0031] Jones PA , Issa J‐PJ , Baylin S (2016) Targeting the cancer epigenome for therapy. Nat Rev Genet 17: 630–641 27629931 10.1038/nrg.2016.93

[embj2019101564-bib-0032] Joshi AA , Struhl K (2005) Eaf3 chromodomain interaction with methylated H3‐K36 links histone deacetylation to Pol II elongation. Mol Cell 20: 971–978 16364921 10.1016/j.molcel.2005.11.021

[embj2019101564-bib-0033] Kadosh D , Struhl K (1998) Histone deacetylase activity of Rpd3 is important for transcriptional repression in vivo. Genes Dev 12: 797–805 9512514 10.1101/gad.12.6.797 PMC316629

[embj2019101564-bib-0034] Kemmeren P , Sameith K , van de Pasch LAL , Benschop JJ , Lenstra TL , Margaritis T , O'Duibhir E , Apweiler E , van Wageningen S , Ko CW *et al* (2014) Large‐scale genetic perturbations reveal regulatory networks and an abundance of gene‐specific repressors. Cell 157: 740–752 24766815 10.1016/j.cell.2014.02.054

[embj2019101564-bib-0035] Keogh M‐C , Kurdistani SK , Morris SA , Ahn SH , Podolny V , Collins SR , Schuldiner M , Chin K , Punna T , Thompson NJ *et al* (2005) Cotranscriptional Set2 methylation of histone H3 lysine 36 recruits a repressive Rpd3 complex. Cell 123: 593–605 16286008 10.1016/j.cell.2005.10.025

[embj2019101564-bib-0036] Kim W , Kim R , Park G , Park J‐W , Kim J‐E (2012) Deficiency of H3K79 histone methyltransferase Dot1‐like protein (DOT1L) inhibits cell proliferation. J Biol Chem 287: 5588–5599 22190683 10.1074/jbc.M111.328138 PMC3285333

[embj2019101564-bib-0037] Kueng S , Oppikofer M , Gasser SM (2013) SIR proteins and the assembly of silent chromatin in budding yeast. Annu Rev Genet 47: 275–306 24016189 10.1146/annurev-genet-021313-173730

[embj2019101564-bib-0038] Kuntimaddi A , Achille NJ , Thorpe J , Lokken AA , Singh R , Hemenway CS , Adli M , Zeleznik‐Le NJ , Bushweller JH (2015) Degree of recruitment of DOT1L to MLL‐AF9 defines level of H3K79 Di‐ and Tri‐methylation on target genes and transformation potential. Cell Rep 11: 808–820 25921540 10.1016/j.celrep.2015.04.004 PMC4426023

[embj2019101564-bib-0039] Lagger S , Meunier D , Mikula M , Brunmeir R , Schlederer M , Artaker M , Pusch O , Egger G , Hagelkruys A , Mikulits W *et al* (2010) Crucial function of histone deacetylase 1 for differentiation of teratomas in mice and humans. EMBO J 29: 3992–4007 20967026 10.1038/emboj.2010.264 PMC3020644

[embj2019101564-bib-0040] Lardenois A , Stuparevic I , Liu Y , Law MJ , Becker E , Smagulova F , Waern K , Guilleux M‐H , Horecka J , Chu A *et al* (2015) The conserved histone deacetylase Rpd3 and its DNA binding subunit Ume6 control dynamic transcript architecture during mitotic growth and meiotic development. Nucleic Acids Res 43: 115–128 25477386 10.1093/nar/gku1185 PMC4288150

[embj2019101564-bib-0041] Lee S , Oh S , Jeong K , Jo H , Choi Y , Seo HD , Kim M , Choe J , Kwon CS , Lee D (2018) Dot1 regulates nucleosome dynamics by its inherent histone chaperone activity in yeast. Nat Commun 9: 240 29339748 10.1038/s41467-017-02759-8 PMC5770421

[embj2019101564-bib-0042] Lenstra TL , Benschop JJ , Kim T , Schulze JM , Brabers NACH , Margaritis T , van de Pasch LAL , van Heesch SAAC , Brok MO , Groot Koerkamp MJA *et al* (2011) The specificity and topology of chromatin interaction pathways in yeast. Mol Cell 42: 536–549 21596317 10.1016/j.molcel.2011.03.026 PMC4435841

[embj2019101564-bib-0043] Leroy G , Dimaggio PA , Chan EY , Zee BM , Blanco MA , Bryant B , Flaniken IZ , Liu S , Kang Y , Trojer P *et al* (2013) A quantitative atlas of histone modification signatures from human cancer cells. Epigenetics Chromatin 6: 20 23826629 10.1186/1756-8935-6-20 PMC3710262

[embj2019101564-bib-0044] Li B , Carey M , Workman JL (2007) The role of chromatin during transcription. Cell 128: 707–719 17320508 10.1016/j.cell.2007.01.015

[embj2019101564-bib-0045] Li Y , Wen H , Xi Y , Tanaka K , Wang H , Peng D , Ren Y , Jin Q , Dent SYR , Li W *et al* (2014) AF9 YEATS domain links histone acetylation to DOT1L‐mediated H3K79 methylation. Cell 159: 558–571 25417107 10.1016/j.cell.2014.09.049 PMC4344132

[embj2019101564-bib-0046] Magraner‐Pardo L , Pelechano V , Coloma MD , Tordera V (2014) Dynamic remodeling of histone modifications in response to osmotic stress in *Saccharomyces cerevisiae* . BMC Genom 15: 247 10.1186/1471-2164-15-247 PMC3986647 24678875

[embj2019101564-bib-0047] McDaniel SL , Strahl BD (2017) Shaping the cellular landscape with Set2/SETD2 methylation. Cell Mol Life Sci 74: 3317–3334 28386724 10.1007/s00018-017-2517-x PMC5545052

[embj2019101564-bib-0048] McKnight JN , Boerma JW , Breeden LL , Tsukiyama T (2015) Global promoter targeting of a conserved lysine deacetylase for transcriptional shutoff during quiescence entry. Mol Cell 59: 732–743 26300265 10.1016/j.molcel.2015.07.014 PMC4560983

[embj2019101564-bib-0049] McKnight JN , Boerma JW , Breeden LL , Tsukiyama T (2015) Gene Expression Omnibus GSE67151 (https://www.ncbi.nlm.nih.gov/geo/query/acc.cgi?acc=GSE67151). [DATASET]

[embj2019101564-bib-0050] Murray SC , Haenni S , Howe FS , Fischl H , Chocian K , Nair A , Mellor J (2015) Sense and antisense transcription are associated with distinct chromatin architectures across genes. Nucleic Acids Res 43: 7823–7837 26130720 10.1093/nar/gkv666 PMC4652749

[embj2019101564-bib-0051] Onder TT , Kara N , Cherry A , Sinha AU , Zhu N , Bernt KM , Cahan P , Marcarci BO , Unternaehrer J , Gupta PB *et al* (2012) Chromatin‐modifying enzymes as modulators of reprogramming. Nature 483: 598–602 22388813 10.1038/nature10953 PMC3501145

[embj2019101564-bib-0052] Radman‐Livaja M , Verzijlbergen KF , Weiner A , van Welsem T , Friedman N , Rando OJ , van Leeuwen F (2011) Patterns and mechanisms of ancestral histone protein inheritance in budding yeast. PLoS Biol 9: e1001075 21666805 10.1371/journal.pbio.1001075 PMC3110181

[embj2019101564-bib-0053] Rando OJ , Winston F (2012) Chromatin and transcription in yeast. Genetics 190: 351–387 22345607 10.1534/genetics.111.132266 PMC3276623

[embj2019101564-bib-0054] Ritchie ME , Phipson B , Wu D , Hu Y , Law CW , Shi W , Smyth GK (2015) limma powers differential expression analyses for RNA‐sequencing and microarray studies. Nucleic Acids Res 43: e47. 25605792 10.1093/nar/gkv007 PMC4402510

[embj2019101564-bib-0055] Rundlett SE , Carmen AA , Suka N , Turner BM , Grunstein M (1998) Transcriptional repression by UME6 involves deacetylation of lysine 5 of histone H4 by RPD3. Nature 392: 831–835 9572144 10.1038/33952

[embj2019101564-bib-0056] Sabra M , Texier P , El Maalouf J , Lomonte P (2013) The Tudor protein survival motor neuron (SMN) is a chromatin‐binding protein that interacts with methylated lysine 79 of histone H3. J Cell Sci 126: 3664–3677 23750013 10.1242/jcs.126003

[embj2019101564-bib-0057] Sadeh R , Launer‐Wachs R , Wandel H , Rahat A , Friedman N (2016) Elucidating combinatorial chromatin states at single‐nucleosome resolution. Mol Cell 63: 1080–1088 27496019 10.1016/j.molcel.2016.07.023 PMC5031555

[embj2019101564-bib-0058] Schulze JM , Jackson J , Nakanishi S , Gardner JM , Hentrich T , Haug J , Johnston M , Jaspersen SL , Kobor MS , Shilatifard A (2009) Linking cell cycle to histone modifications: SBF and H2B monoubiquitination machinery and cell‐cycle regulation of H3K79 dimethylation. Mol Cell 35: 626–641 19682934 10.1016/j.molcel.2009.07.017 PMC3222332

[embj2019101564-bib-0059] Schulze JM , Hentrich T , Nakanishi S , Gupta A , Emberly E , Shilatifard A , Kobor MS (2011) Splitting the task: Ubp8 and Ubp10 deubiquitinate different cellular pools of H2BK123. Genes Dev 25: 2242–2247 22056669 10.1101/gad.177220.111 PMC3219228

[embj2019101564-bib-0060] Shortt J , Ott CJ , Johnstone RW , Bradner JE (2017) A chemical probe toolbox for dissecting the cancer epigenome. Nat Rev Cancer 17: 160–183 28228643 10.1038/nrc.2016.148

[embj2019101564-bib-0061] Soria‐Valles C , Osorio FG , Gutiérrez‐Fernández A , De Los Angeles A , Bueno C , Menéndez P , Martín‐Subero JI , Daley GQ , Freije JMP , López‐Otín C (2015) NF‐κB activation impairs somatic cell reprogramming in ageing. Nat Cell Biol 17: 1004–1013 26214134 10.1038/ncb3207

[embj2019101564-bib-0062] Stein EM , Tallman MS (2016) Emerging therapeutic drugs for AML. Blood 127: 71–78 26660428 10.1182/blood-2015-07-604538 PMC4915807

[embj2019101564-bib-0063] Stein EM , Garcia‐Manero G , Rizzieri DA , Tibes R , Berdeja JG , Savona MR , Jongen‐Lavrenic M , Altman JK , Thomson B , Blakemore SJ *et al* (2018) The DOT1L inhibitor pinometostat reduces H3K79 methylation and has modest clinical activity in adult acute leukemia. Blood 131: 2661–2669 29724899 10.1182/blood-2017-12-818948 PMC6265654

[embj2019101564-bib-0064] Stulemeijer IJ , Pike BL , Faber AW , Verzijlbergen KF , van Welsem T , Frederiks F , Lenstra TL , Holstege FC , Gasser SM , van Leeuwen F (2011) Dot1 binding induces chromatin rearrangements by histone methylation‐dependent and ‐independent mechanisms. Epigenetics Chromatin 4: 2 21291527 10.1186/1756-8935-4-2 PMC3038881

[embj2019101564-bib-0065] Sun Z‐W , Hampsey M (1999) A general requirement for the Sin3‐Rpd3 histone deacetylase complex in regulating silencing in *Saccharomyces cerevisiae* . Genetics 152: 921–932 10388812 10.1093/genetics/152.3.921 PMC1460667

[embj2019101564-bib-0066] Teixeira MC , Monteiro PT , Guerreiro JF , Gonçalves JP , Mira NP , dos Santos SC , Cabrito TR , Palma M , Costa C , Francisco AP *et al* (2014) The YEASTRACT database: an upgraded information system for the analysis of gene and genomic transcription regulation in *Saccharomyces cerevisiae* . Nucleic Acids Res 42: D161–D166 24170807 10.1093/nar/gkt1015 PMC3965121

[embj2019101564-bib-0067] Thurtle‐Schmidt DM , Dodson AE , Rine J (2016) Histone deacetylases with antagonistic roles in saccharomyces cerevisiae heterochromatin formation. Genetics 204: 177–190 27489001 10.1534/genetics.116.190835 PMC5012384

[embj2019101564-bib-0068] Van Leeuwen F , Gottschling DE (2002) Assays for gene silencing in yeast. Methods Enzymol 350: 165–186 12073311 10.1016/s0076-6879(02)50962-9

[embj2019101564-bib-0069] Van Welsem T , Korthout T , Ekkebus R , Morais D , Molenaar TM , van Harten K , Poramba‐Liyanage DW , Sun SM , Lenstra TL , Srivas R *et al* (2018) Dot1 promotes H2B ubiquitination by a methyltransferase‐independent mechanism. Nucleic Acids Res 46: 11251–11261 30203048 10.1093/nar/gky801 PMC6265471

[embj2019101564-bib-0070] Venkatesh S , Workman JL , Smolle M (2013) UpSETing chromatin during non‐coding RNA production. Epigenetics Chromatin 6: 16 23738864 10.1186/1756-8935-6-16 PMC3680234

[embj2019101564-bib-0071] Verzijlbergen KF , Menendez‐Benito V , van Welsem T , van Deventer SJ , Lindstrom DL , Ovaa H , Neefjes J , Gottschling DE , van Leeuwen F (2010) Recombination‐induced tag exchange to track old and new proteins. Proc Natl Acad Sci USA 107: 64–68 20018668 10.1073/pnas.0911164107 PMC2806724

[embj2019101564-bib-0072] Vlaming H , van Welsem T , de Graaf EL , Ontoso D , Altelaar AM , San‐Segundo PA , Heck AJ , Van Leeuwen F (2014) Flexibility in crosstalk between H2B ubiquitination and H3 methylation in vivo. EMBO Rep 15: 1077–1084 25141862 10.15252/embr.201438793 PMC4253848

[embj2019101564-bib-0073] Vlaming H , Van Leeuwen F (2016) The upstreams and downstreams of H3K79 methylation by DOT1L. Chromosoma 125: 593–605 26728620 10.1007/s00412-015-0570-5

[embj2019101564-bib-0074] Vlaming H , Molenaar TM , van Welsem T , Poramba‐Liyanage DW , Smith DE , Velds A , Hoekman L , Korthout T , Hendriks S , Altelaar AM *et al* (2016) Direct screening for chromatin status on DNA barcodes in yeast delineates the regulome of H3K79 methylation by Dot1. Elife 5: e18919 27922451 10.7554/eLife.18919 PMC5179194

[embj2019101564-bib-0075] Wan L , Wen H , Li Y , Lyu J , Xi Y , Hoshii T , Joseph JK , Wang X , Loh Y‐HE , Erb MA *et al* (2017) ENL links histone acetylation to oncogenic gene expression in acute myeloid leukaemia. Nature 543: 265–269 28241141 10.1038/nature21687 PMC5372383

[embj2019101564-bib-0076] Wang X , Chen C‐W , Armstrong SA (2016) The role of DOT1L in the maintenance of leukemia gene expression. Curr Opin Genet Dev 36: 68–72 27151433 10.1016/j.gde.2016.03.015

[embj2019101564-bib-0077] Waters NJ , Daigle SR , Rehlaender BN , Basavapathruni A , Campbell CT , Jensen TB , Truitt BF , Olhava EJ , Pollock RM , Stickland KA *et al* (2015) Exploring drug delivery for the DOT1L inhibitor pinometostat (EPZ‐5676): Subcutaneous administration as an alternative to continuous IV infusion, in the pursuit of an epigenetic target. J Control Release 220: 758–765 26385168 10.1016/j.jconrel.2015.09.023

[embj2019101564-bib-0078] Weake VM , Workman JL (2008) Histone ubiquitination: triggering gene activity. Mol Cell 29: 653–663 18374642 10.1016/j.molcel.2008.02.014

[embj2019101564-bib-0079] Weinberger L , Voichek Y , Tirosh I , Hornung G , Amit I , Barkai N (2012) Expression noise and acetylation profiles distinguish HDAC functions. Mol Cell 47: 193–202 22683268 10.1016/j.molcel.2012.05.008 PMC3408861

[embj2019101564-bib-0080] Weiner A , Hsieh T‐HSS , Appleboim A , Chen HVV , Rahat A , Amit I , Rando OJJ , Friedman N (2015) High‐resolution chromatin dynamics during a yeast stress response. Mol Cell 58: 371–386 25801168 10.1016/j.molcel.2015.02.002 PMC4405355

[embj2019101564-bib-0081] West AC , Johnstone RW (2014) New and emerging HDAC inhibitors for cancer treatment. J Clin Invest 124: 30–39 24382387 10.1172/JCI69738 PMC3871231

[embj2019101564-bib-0082] Wood K , Tellier M , Murphy S (2018) DOT1L and H3K79 methylation in transcription and genomic stability. Biomolecules 8: 11 10.3390/biom8010011 PMC5871980 29495487

[embj2019101564-bib-0083] Xue Y , Van C , Pradhan SK , Su T , Gehrke J , Kuryan BG , Kitada T , Vashisht A , Tran N , Wohlschlegel J *et al* (2015) The Ino80 complex prevents invasion of euchromatin into silent chromatin. Genes Dev 29: 350–355 25691465 10.1101/gad.256255.114 PMC4335291

[embj2019101564-bib-0084] Xue Y , Van C , Pradhan SK , Su T , Gehrke J , Kuryan BG , Kitada T , Vashisht A , Tran N , Wohlschlegel J *et al* (2015) Gene Expression Omnibus GSE52000 (https://www.ncbi.nlm.nih.gov/geo/query/acc.cgi?acc=GSE52000). [DATASET]

[embj2019101564-bib-0085] Xue Y , Pradhan SK , Sun F , Chronis C , Tran N , Su T , Van C , Vashisht A , Wohlschlegel J , Peterson CL *et al* (2017) Mot 1, Ino80C, and NC2 function coordinately to regulate pervasive transcription in yeast and mammals. Mol Cell 67: 594–607.e4 28735899 10.1016/j.molcel.2017.06.029 PMC5573681

[embj2019101564-bib-0086] Yang X‐J , Seto E (2008) The Rpd3/Hda1 family of lysine deacetylases: from bacteria and yeast to mice and men. Nat Rev Mol Cell Biol 9: 206–218 18292778 10.1038/nrm2346 PMC2667380

[embj2019101564-bib-0087] Zhang L , Deng L , Chen F , Yao Y , Wu B , Wei L , Mo Q , Song Y (2014) Inhibition of histone H3K79 methylation selectively inhibits proliferation, self‐renewal and metastatic potential of breast cancer. Oncotarget 5: 10665–10677 25359765 10.18632/oncotarget.2496 PMC4279401

[embj2019101564-bib-0088] Zhang T , Cooper S , Brockdorff N (2015) The interplay of histone modifications – writers that read. EMBO Rep 16: 1467–1481 26474904 10.15252/embr.201540945 PMC4641500

[embj2019101564-bib-0089] Zhou L , Holt MT , Ohashi N , Zhao A , Müller MM , Wang B , Muir TW (2016) Evidence that ubiquitylated H2B corrals hDot1L on the nucleosomal surface to induce H3K79 methylation. Nat Commun 7: 10589 26830124 10.1038/ncomms10589 PMC4740876

