# Supplementary material for: Conserved crosstalk between histone deacetylation and H3K79 methylation generates DOT1L‐dose dependency in HDAC1‐deficient thymic lymphoma
Source: EMBO J. 2019 Jun 17;38(14):e101564. doi: 10.15252/embj.2019101564 (PMC6627229; doi:10.15252/embj.2019101564)
Supplement: Supplementary file 1 — Appendix [file EMBJ-38-e101564-s001.pdf]

Appendix

Appendix Table S1 Yeast strains used in this study
Appendix Table S2 Primers used for qPCR
Appendix Supplementary Methods
Appendix Supplementary References

Appendix Table S1 Yeast strains used in this study

Strain name	Genotype	Reference	Figures
NatMX-KO x Barcoders library	MATa can1Δ::STE2pr-Sphis5 lyp1Δ his3Δ1 leu2Δ0 ura3Δ0 met15Δ0 hoΔ::barcodedKanMX GOI::NatMX	(Vlaming *et al*, 2016)	1B
NKI4560	MATa can1Δ::STE2pr-Sp_his5 lyp1Δ his3Δ1 leu2Δ0 ura3Δ0 met15Δ0 hoΔ::barcode(0001)KanMX	(Vlaming *et al*, 2016)	1C, EV1A
NKI4557	MATa can1Δ::STE2pr-Sp_his5 lyp1Δ his3Δ1 leu2Δ0 ura3Δ0 met15Δ0 hoΔ::barcode(sir2)KanMX dot1Δ::NatMX	(Vlaming *et al*, 2016)	S1A
NKI4558	MATa can1Δ::STE2pr-Sp_his5 lyp1Δ his3Δ1 leu2Δ0 ura3Δ0 met15Δ0 hoΔ::barcode(sir3)KanMX bre1Δ::NatMX	(Vlaming *et al*, 2016)	S1A
NKI4643	MATa can1Δ::STE2pr-Sp_his5 lyp1Δ his3Δ1 leu2Δ0 ura3Δ0 met15Δ0 hoΔ::barcode(0791)KanMX rpd3Δ::NatMX	From library	1C, EV1A
NKI4644	MATa can1Δ::STE2pr-Sp_his5 lyp1Δ his3Δ1 leu2Δ0 ura3Δ0 met15Δ0 hoΔ::barcode(1002)KanMX sin3Δ::NatMX	From library	1C, EV1A
NKI4657	MATa his3Δ200 leu2Δ0 trp1Δ63 ura3Δ0 met15Δ0 hoΔ:: barcode(sir4)KanMX	(Vlaming *et al*, 2016)	All ChIPs
NKI4713	MATa his3Δ200 leu2Δ0 trp1Δ63 ura3Δ0 met15Δ0 hoΔ:: barcode(sir4)KanMX rpd3Δ::NatMX	This study	All ChIPs

Appendix Table S2 Primers used for qPCR

Primer	Species	Position[a]	Sequence
Gapdh qPCR - F	mouse		CATCTTCTTGTGCAGTGCCAG
Gapdh_qPCR_R	mouse		GTGAGTGGAGTCATACTGGAACA
Dot1L Exons5-7 qPCR Fw	mouse		CAGAGGATGACCTGTTTGTCG
Dot1L Exons5-7 qPCR Rv	mouse		CATCCACTTCCTGAACTCTCG
SPA2_High_Qfor	budding yeast	379	ATCAAGAGAAGAGGGTTCGACAAG
SPA2_High_Qrev	budding yeast	379	CATCGGCTGCGGTAATGG
PCH2_ORF_Qfor	budding yeast	176	CTGACTCGAAACAAAACAGCA
PCH2_ORF_Qrev	budding yeast	176	CTTCCTTGCCCCTCTCTCAT
SIP4_ORF_Qfor	budding yeast	268	CTCTGTCAGAAAGGCGCATG
SIP4_ORF_Qrev	budding yeast	268	CGCTGGAACTCGCATTCATA
DBP1_ORF_Qfor	budding yeast	254	CTGGAAGGCAAACTGGGAAC
DBP1_ORF_Qrev	budding yeast	254	TAGGCCCCGGTATATGCTTG
ZIP1_ORF_Qfor	budding yeast	254	ACCCCACAAAACTTCTACCGA
ZIP1_ORF_Qrev	budding yeast	254	TTTCAATTGCGGCAACATCA
IRC7_Qfor1	budding yeast	204	CAGCAGGTTCTCCAACTATTGACA
IRC7_Qrev1	budding yeast	204	CCAGCACCGCCGGTTA
NoORF_Qfor	budding yeast		GGCTGTCAGAATATGGGGCCGTAGTA
NoORF_Qrev	budding yeast		CACCCCGAAGCTGCTTTCACAATAC

[a] distance of PCR product from start of coding sequence

Appendix Supplementary Methods

Mouse generation and crosses

The Dot1l$^{tm1a(KOMP)Wtsi}$ mouse was generated by the Wellcome Trust Sanger Institute (WTSI) and obtained from the KOMP Repository (www.komp.org) (Skarnes *et al*, 2011). Since this mouse had a knock-out first allele, FLPe in a C57BL/6 background was crossed in to remove the FRT-flanked cassette (B6.Cg-Tg(ACTFLPe)9205Dym/A, MGI:2448985; Rodríguez *et al,* 2000). FLPe was crossed out to obtain C57BL/6 *Dot1L*$^{f/f}$ mice, with LoxP sites flanking exon 2 of *Dot1L*. No thymic lymphomas were observed in C57BL/6 *Lck*-Cre;*Dot1L*$^{f/f}$ mice. For this study, mice with the conditional *Dot1L* allele were crossed with mice bearing *Lck*-Cre and conditional Hdac1 alleles, which were described before (Heideman *et al*, 2013). All mice analyzed in this study were progeny of this cross and were in a mixed FVB/n, C57BL/6, and 129/Sv background. Wild-type control mice were the *Lck*-Cre-negative littermates of the other mice used in this study.

Nuclear extract preparation and immunoblotting

Thymic lymphoma cell lines were collected and washed by PBS. Single-cell suspensions of thymuses were obtained by passing the tissues through a 70 µm cell strainer, and cells were pelleted and washed with PBS. Samples were kept cold at all times and all buffers were supplemented with Complete protease inhibitors (Roche), Trichostatin A and nicotinamide. To make nuclear extracts, cells were first incubated in hypotonic lysis buffer (10mM Tris (pH 7.8), 5 mM $MgCl_2$, 10 mM KCl, 0.1 mM EDTA, 300 mM sucrose, 5 mM B-glycerol) for ten minutes. Nonidet P-40 was added to an end concentration of 0.12% to rupture the cells. Nuclei were collected by centrifugation and lysed in RIPA buffer (20mM Tris (pH 7.5), 150mM NaCl, 1% Nonidet P-40, 0.5% sodium deoxycholate, 1mM EDTA, 0.1% SDS) for 30 minutes. All buffers were supplemented with Complete protease inhibitors (Roche), Trichostatin A and nicotinamide. Samples were sonicated for 2.5 minutes (10 second pulses) using the Diagenode Biorupter to solubilize chromatin. After this step, debris was pelleted and the supernatant was collected. Protein concentration was determined using the *DC* protein assay (Bio-Rad). The immunoblotting procedure was as described in (Vlaming *et al*, 2014). Yeast extracts were loaded on 16% polyacrylamide gels; murine extracts were loaded on gradient gels (4-12% Bis-Tris NuPAGE mini gels).

Antibodies

Blots with yeast samples were probed with antibodies against Dot1 (RRID: AB_2631109; Van Leeuwen *et al*, 2002), Pgk1 (A-6457, Invitrogen) and H2B (39238, Active Motif). Blots with mouse samples were probed with antibodies against HDAC1 (NB100-56340, Novus Biologicals), H3K79me1 and H3K79me2 (RRID:AB_2631105 and AB_2631106; Frederiks *et al*, 2008), H2BK120ub (#5546, Cell Signaling Technology), H3K9ac (ab4441, Abcam), total H3 (ab1791, Abcam), and a newly generated H4 antibody. Rabbit polyclonal antibodies against histone H4 were generated by immunizing with the peptide (C)VYALKRQGRTLYGFG of the C terminus of histone H4 of *S. cerevisiae*. The polyclonal serum recognizes human and mouse histone H4. ChIP experiments were performed using antibodies against H3K79me1, H3K79me3 and total H3 (RRID:AB_2631105, AB_2631107, AB_2631108; Frederiks *et al*, 2008) and antibodies against H2B (39238, Active Motif) and a new site-specific antibody against yeast H2Bub that we recently developed (Vlaming *et al*, 2016). For immunohistochemistry, antibodies against HDAC1 (ab31263; Abcam) and H3K79me2 (RRID:AB_2631106; Frederiks *et al*, 2008) were used.

ChIP-seq library preparation and data analysis

Library preparation and sequencing were performed by the NKI Genomics Core Facility, in two batches. Libraries from the ChIP samples from WT #1 were prepared using the TruSeq® DNA LT Sample Preparation kit (Illumina, cat no. FC-121-2001), using ten times less adapter in the adapter ligation step. After fifteen PCR cycles, a size selection cleanup was performed using 0.5X Agencourt

AMPure XP PCR Purification beads (Beckman Coulter, cat no A63881) to get rid of large fragments due to crosslinking DNA. The supernatant of the 0.5X cleanup was used to catch the smaller fragments; this supernatant was cleaned up 2 times with 1X beads to remove primers present in the libraries. Samples were pooled equimolarly and subjected to sequencing on an Illumina HiSeq2000 machine in a single-red 50bp run. Libraries from the ChIP samples from WT #2 and two *rpd3Δ* replicates were prepared using the KAPA HTP Library Preparation Kit, Illumina® platforms (KAPA Biosystems KK8234), using Illumina-provided adapters at 200nM. After eleven PCR cycles, cleanup and pooling was as described above. Samples were sequenced in a single-read 65bp run on an Illumina HiSeq2500 machine. ChIP libraries from an *sin3Δ* strain prepared in the first batch (with WT #1) gave results comparable to the two *rpd3Δ* replicates from batch two. Reads were mapped to the *Saccharomyces cerevisiae* reference genome R64-2-1 with BWA version 0.6.1 and filtered for mapping quality 37 (Engel *et al*, 2014; Li & Durbin, 2009). Each read was extended to 150 bp. Each sample was normalized for the sequencing depth by converting to Reads per Genomic Content (RPGC) with DeepTools (Ramírez *et al*, 2016). This was done by dividing the coverage by the sequencing depth, calculated as (total number of mapped reads * fragment length) / effective genome size (12.1×10^6 bp). Data from the biological duplicates was found to be similar and the data sets were merged for further analyses.

<u>Epi-ID analysis</u>
Data from the Epi-ID H3K79me regulator screen described in Vlaming *et al* (2016) were used. As described, a growth-corrected methylation score was calculated by first calculating the H3K79me3/H3K79me1 ratio and then subtracting the value expected based on the growth rate of the strain (Vlaming *et al*, 2016). The CLIK tool (Dittmar *et al*, 2013) was used to define groups of candidate regulators, and to determine the enrichment of the Rpd3L complex. Data on all components of Rpd3L and Rpd3S was obtained in the screen. Deletions were checked by PCR and barcodes were checked by Sanger sequencing. All deletions could be confirmed, with the exception of *sds3Δ*, which was eliminated from the plot in Figure 1B.

Appendix Supplementary References

Dittmar JC, Pierce S, Rothstein R & Reid RJD (2013) Physical and genetic-interaction density reveals functional organization and informs significance cutoffs in genome-wide screens. *Proc. Natl. Acad. Sci.* **110:** 7389–7394

Engel SR, Dietrich FS, Fisk DG, Binkley G, Balakrishnan R, Costanzo MC, Dwight SS, Hitz BC, Karra K, Nash RS, Weng S, Wong ED, Lloyd P, Skrzypek MS, Miyasato SR, Simison M & Cherry JM (2014) The Reference Genome Sequence of Saccharomyces cerevisiae: Then and Now. *G3* **4:** 389–398

Frederiks F, Tzouros M, Oudgenoeg G, van Welsem T, Fornerod M, Krijgsveld J & van Leeuwen F (2008) Nonprocessive methylation by Dot1 leads to functional redundancy of histone H3K79 methylation states. *Nat. Struct. Mol. Biol.* **15:** 550–557

Heideman MR, Wilting RH, Yanover E, Velds A, de Jong J, Kerkhoven RM, Jacobs H, Wessels LF & Dannenberg J-H (2013) Dosage-dependent tumor suppression by histone deacetylases 1 and 2 through regulation of c-Myc collaborating genes and p53 function. *Blood* **121:** 2038–2050

Van Leeuwen F, Gafken PR & Gottschling DE (2002) Dot1p Modulates Silencing in Yeast by Methylation of the Nucleosome Core. *Cell* **109:** 745–756

Li H & Durbin R (2009) Fast and accurate short read alignment with Burrows-Wheeler transform. *Bioinformatics* **25:** 1754–1760

Ramírez F, Ryan DP, Grüning B, Bhardwaj V, Kilpert F, Richter AS, Heyne S, Dündar F & Manke T (2016) deepTools2: a next generation web server for deep-sequencing data analysis. *Nucleic Acids Res.* **44:** W160–W165

Rodríguez CI, Buchholz F, Galloway J, Sequerra R, Kasper J, Ayala R, Stewart AF & Dymecki SM (2000) High-efficiency deleter mice show that FLPe is an alternative to Cre-loxP. *Nat. Genet.* **25:** 139–40

Skarnes WC, Rosen B, West AP, Koutsourakis M, Bushell W, Iyer V, Mujica AO, Thomas M, Harrow J, Cox T, Jackson D, Severin J, Biggs P, Fu J, Nefedov M, de Jong PJ, Stewart AF & Bradley A (2011) A conditional knockout resource for the genome-wide study of mouse gene function. *Nature* **474:** 337–342

Vlaming H, Molenaar TM, van Welsem T, Poramba-Liyanage DW, Smith DE, Velds A, Hoekman L, Korthout T, Hendriks S, Altelaar AM & van Leeuwen F (2016) Direct screening for chromatin status on DNA barcodes in yeast delineates the regulome of H3K79 methylation by Dot1. *Elife* **5:** e18919

Vlaming H, van Welsem T, de Graaf EL, Ontoso D, Altelaar AM, San-Segundo PA, Heck AJ & Van Leeuwen F (2014) Flexibility in crosstalk between H2B ubiquitination and H3 methylation in vivo. *EMBO Rep.* **15:** 1077–1084
