# Supplementary material for: Conserved crosstalk between histone deacetylation and H3K79 methylation generates DOT1L‐dose dependency in HDAC1‐deficient thymic lymphoma
Source: EMBO J. 2019 Jun 17;38(14):e101564. doi: 10.15252/embj.2019101564 (PMC6627229; doi:10.15252/embj.2019101564)
Supplement: Supplementary file 2 — Expanded View Figures PDF [file EMBJ-38-e101564-s002.pdf]

Expanded View Figures

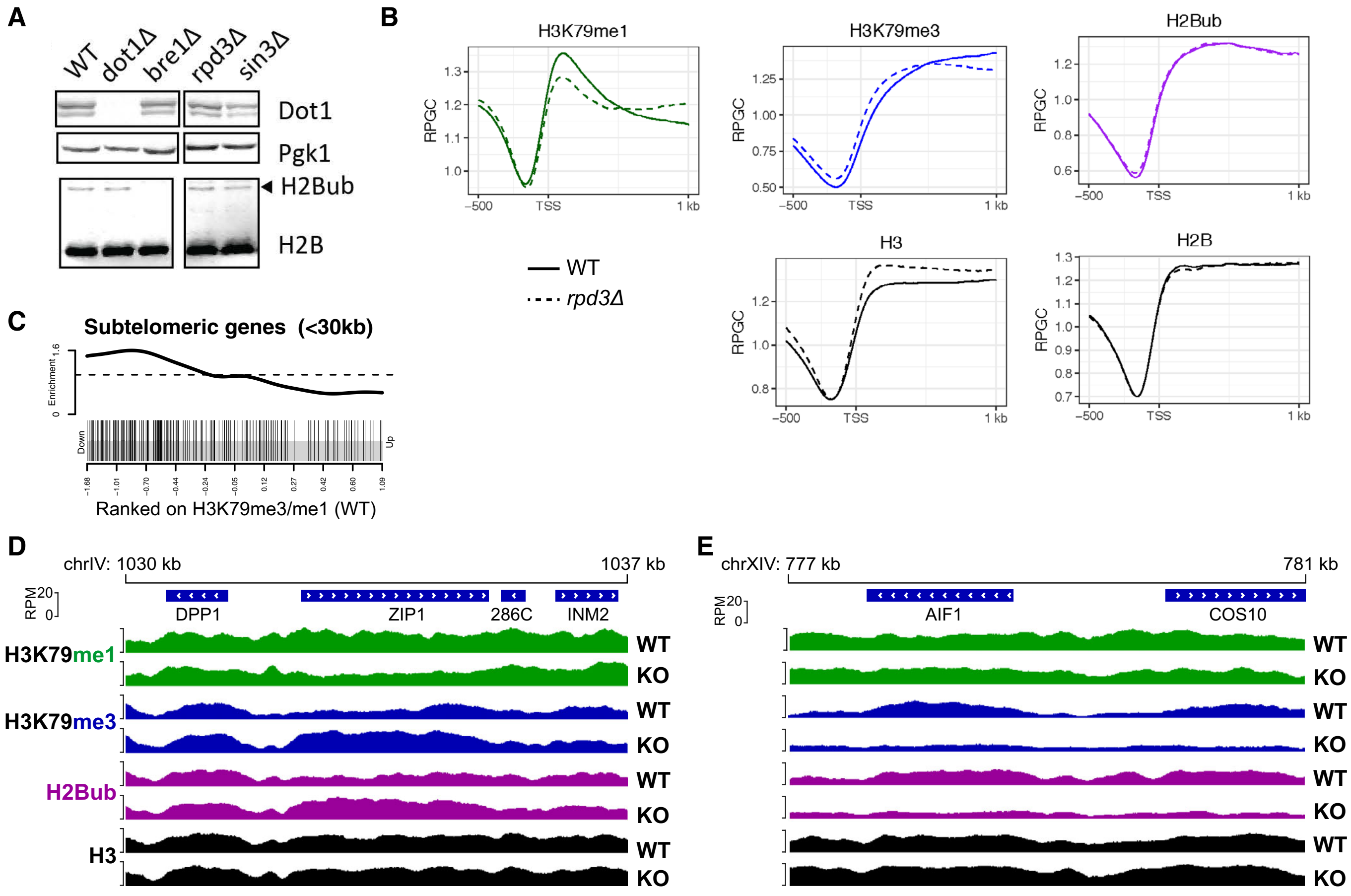

Figure EV1. Rpd3 and Sin3 negatively regulate H3K79 methylation.

A Immunoblots show that deletion of *RPD3* or *SIN3* does not lead to a detectable increase in global H2Bub or Dot1 protein levels.

B Metagene plots of H3K79me1, H3K79me3, H2Bub, H3, and H2B in *rpd3Δ* and wild-type strains.

C Gene set enrichment analysis shows that subtelomeric genes (< 30 kb of telomeres) are enriched among genes with low H3K79 methylation (measured by the average H3K79me3/H3K79me1 ratio in the first 500 bp of the ORF).

D–E Snapshots of depth-normalized ChIP-seq data tracks from wild-type and *rpd3Δ* strains showing 7 kb surrounding meiotic gene *ZIP1* (D) and subtelomeric genes *AIF1* and *COS10* (E). All tracks have the same y-axis (0–20 rpm), which, for comparison, is also the same scale as in Fig 1E.

Source data are available online for this figure.

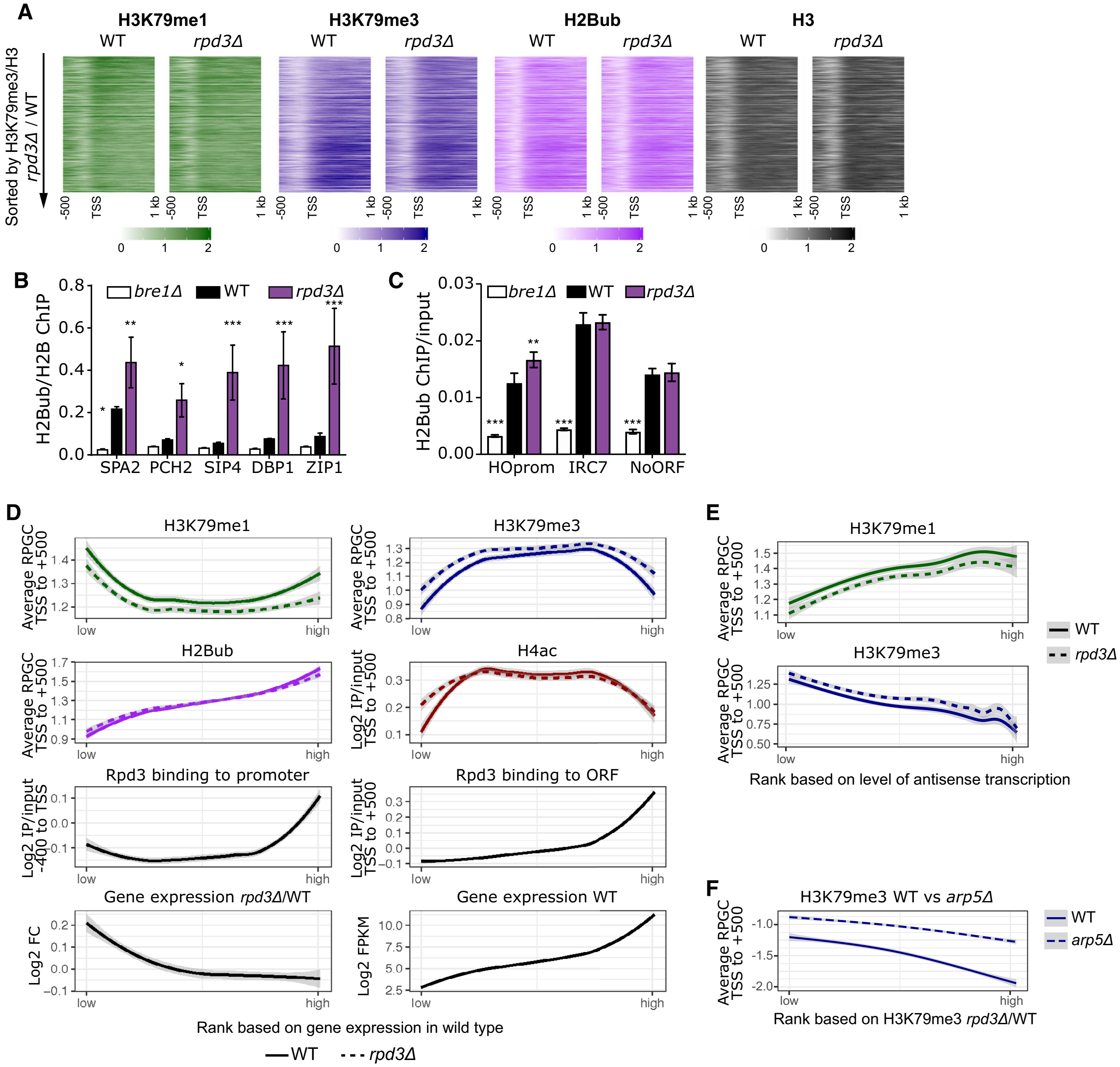

Figure EV2. ChIP-seq and ChIP-qPCR data from WT vs *rpd3Δ* cells, and the relation between transcription and histone modifications.

A Heatmaps of H3K79me1, H3K79me3, H2Bub, and H3 sorted on H3K79me3/H3 *rpd3Δ*/WT.

B H2Bub ChIP-qPCR (H2Bub/H2B) at 5′ ends of indicated genes in wild-type and *rpd3Δ* cells, with *bre1Δ* cells serving as a negative control. Error bars indicate standard deviation of three biological replicates. $*P < 0.05$, $**P < 0.01$, and $***P < 0.001$ by two-way ANOVA, comparison to wild type.

C H2Bub ChIP-qPCR (H2Bub/input) at the subtelomeric IRC7 gene, the promoter of the barcoded HO locus, and a non-transcribed region (NoORF) (Imbeault *et al*, 2008; Verzijlbergen *et al*, 2010). Error bars indicate standard deviation of three biological replicates. $*P < 0.05$, $**P < 0.01$, and $***P < 0.001$ by two-way ANOVA, comparison to wild type.

D ChIP-seq and RNA-seq data per gene (same data as in Fig 2A) ranked on gene expression level in wild-type cells, smoothed using locally weighted regression. The shaded band around the line shows the 95% confidence interval.

E Similar to panel (D), but using a ranking based on the level of antisense transcription per gene in wild-type cells as calculated by Brown *et al* (2018) using data from Churchman and Weissman (2011).

F H3K79me3 ChIP-seq data in gene promoters (−400 to TSS, where the Arp5 effect is maximal) in wild-type and *arp5Δ* cells (Xue *et al*, 2015), ranked by the effect Rpd3 has on H3K79me3 on each gene (same ranking as in Fig 2A).

Source data are available online for this figure.

 © 2019 The Authors

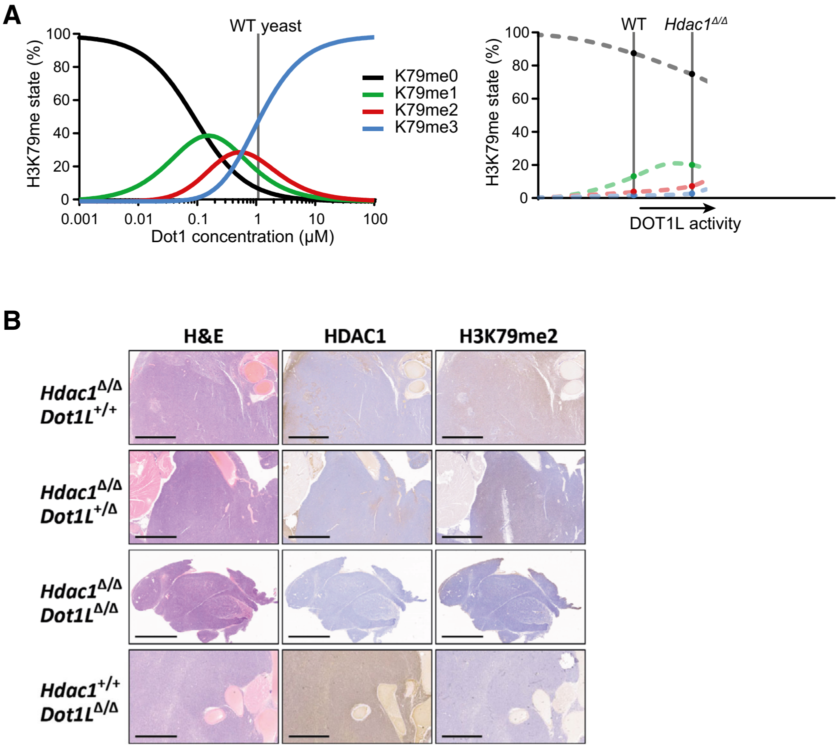

Figure EV3. The distributive nature of Dot1/DOT1L and thymus immunohistochemistry.

A Yeast Dot1 is a distributive enzyme and shows waves of the different methylation states over a range of Dot1 concentrations in yeast (De Vos *et al*, 2011), Fig from De Vos *et al* (2017). The catalytic nature of mammalian DOT1L enzymes is not known, but the observation that the abundance of each methylation state increases upon *Hdac1* deletion does not conflict with a distributive nature. Since H3K79 methylation levels are low in mammalian cells, it is possible we are looking at the start of the methylation waves, as indicated by the dotted lines.

B Representative H&E and immunohistochemical staining of sequential sections of thymic lymphomas of the indicated genotypes. The scale bar represents 2 mm.

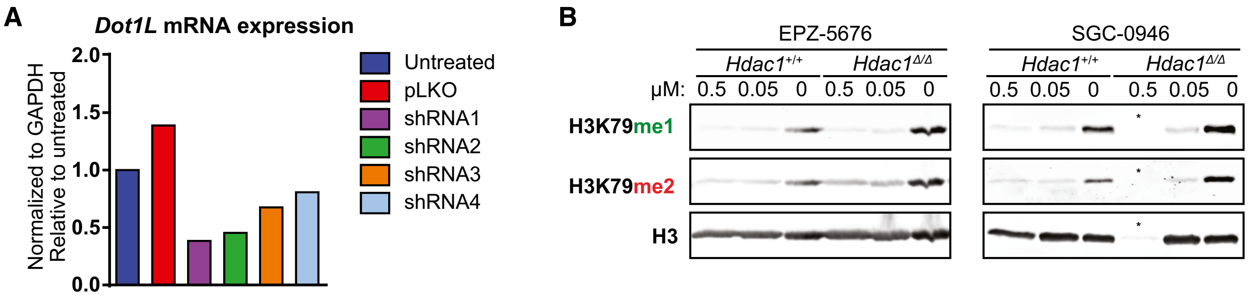

Figure EV4. Control experiments belonging to Fig 4.

A Knockdown efficiency of the shRNAs used in Fig 4D. The shRNAs 1 and 2 give a knockdown of more than 50%.

B Immunoblot analysis of H3K79 methylation levels in the two cell lines with and without inhibitors. Both the effect of the inhibitors and the difference between the cell lines can be observed; * indicates a lane that was underloaded because most cells had died. For cells with EPZ-5676 treatment, the H3K79me1 blot was re-probed with α-H3K79me2, following stripping the previous antibody and confirming that the stripping was complete. Finally, the blot was re-probed with α-H3-C.

Source data are available online for this figure.

© 2019 The Authors
