# Supplementary figures and images for: Conserved crosstalk between histone deacetylation and H3K79 methylation generates DOT1L‐dose dependency in HDAC1‐deficient thymic lymphoma
Source: EMBO J. 2019 Jun 17;38(14):e101564. doi: 10.15252/embj.2019101564 (PMC6627229; doi:10.15252/embj.2019101564)

**Fig EV4B**

**EPZ-5676**

**SGC0946**

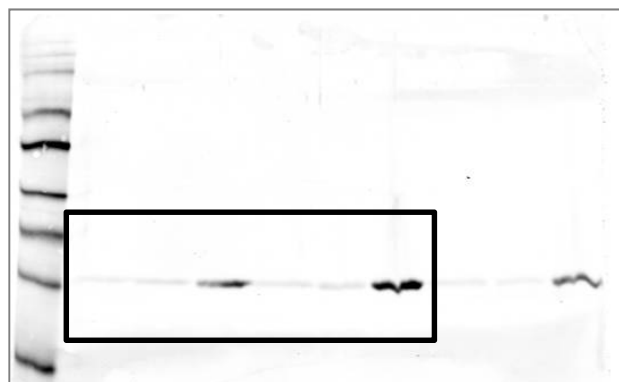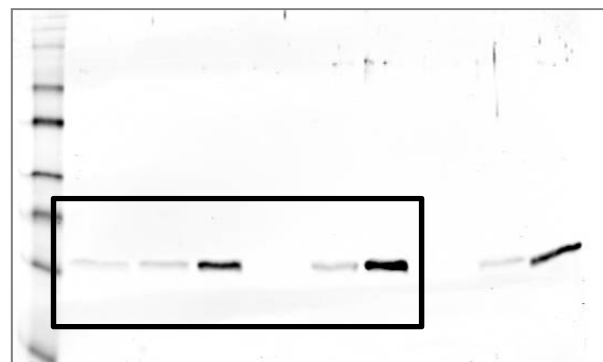

H3K79me1

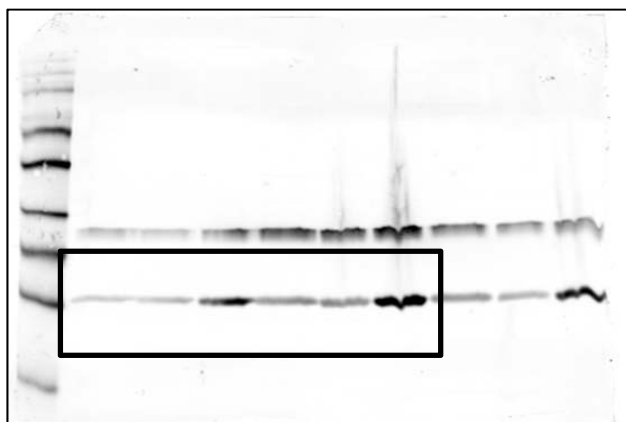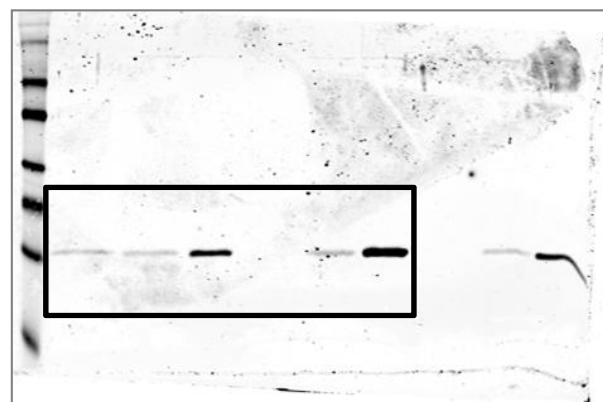

H3K79me2

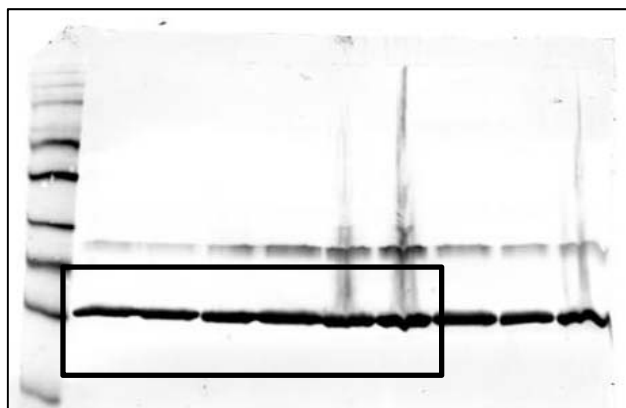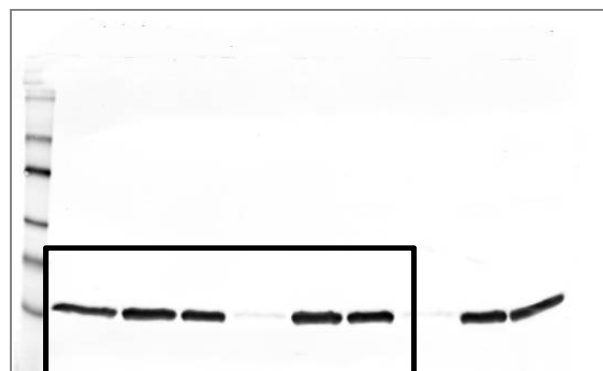

H3c

Supplement: Supplementary file 3 — Source Data for Expanded View [file EMBJ-38-e101564-s008.zip › EMBOJ-2019-101564_SourceDataForFigureEV4B.pdf]

**Dot1**

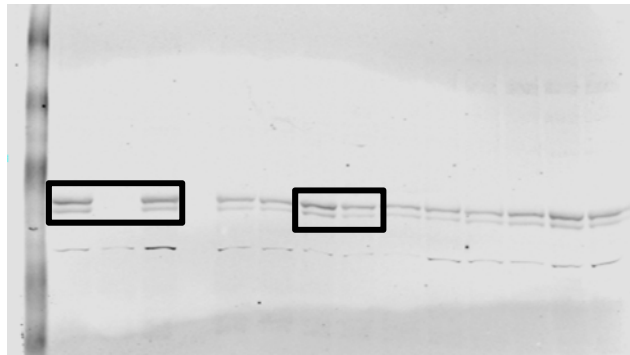

**Pgk1**

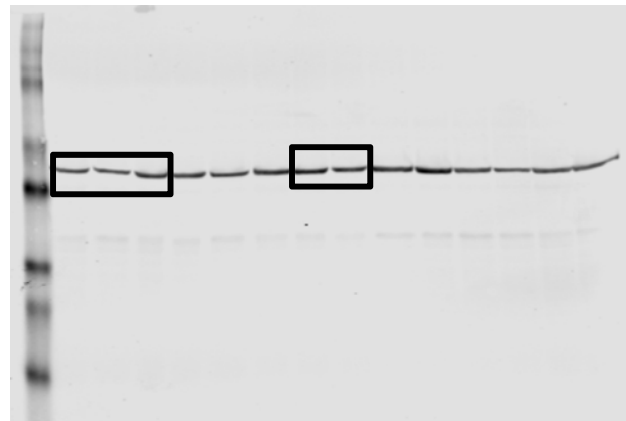

**H2B**

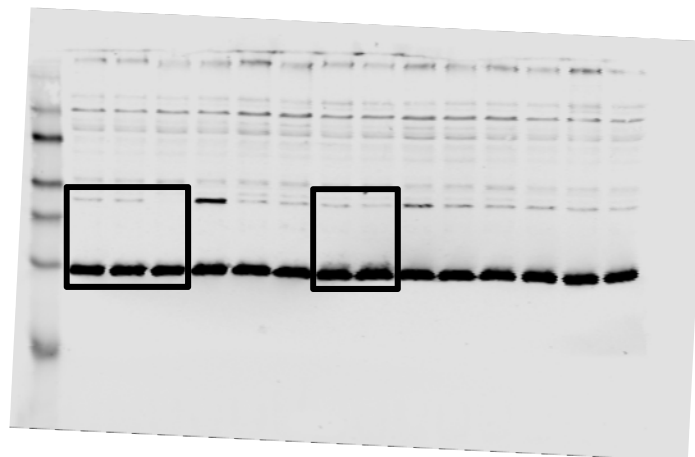

Supplement: Supplementary file 3 — Source Data for Expanded View [file EMBJ-38-e101564-s008.zip › EMBOJ-2019-101564_SourceDataForFigureEV1A.pdf]

Source  
Fig 3B

H&E

HDAC1

H3K79me2

*HDAC1Δ/Δ*  
*DOT1L+/+*

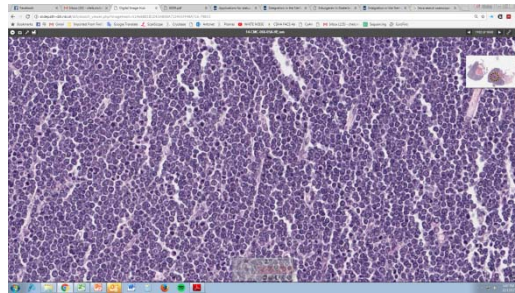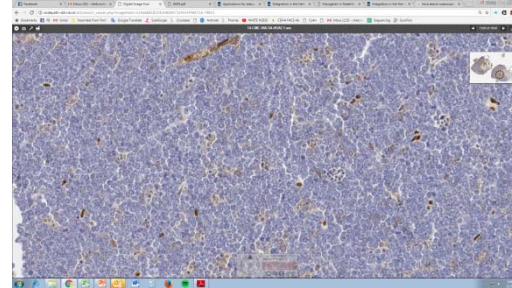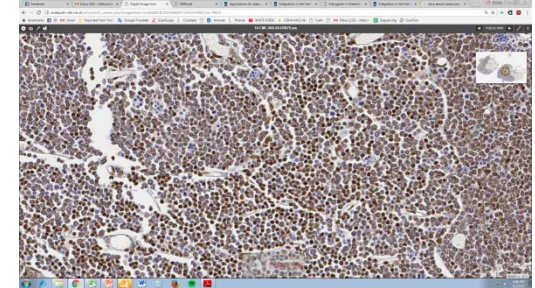

*HDAC1Δ/Δ*  
*DOT1L+/Δ*

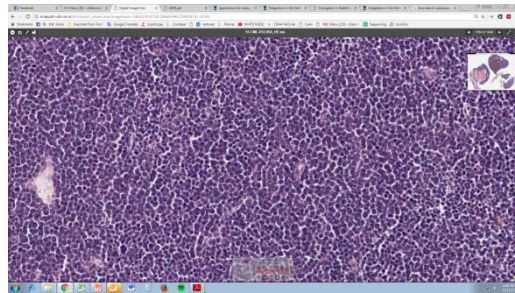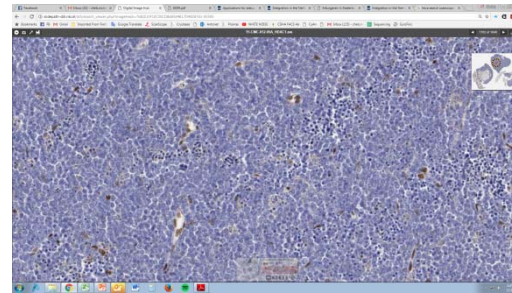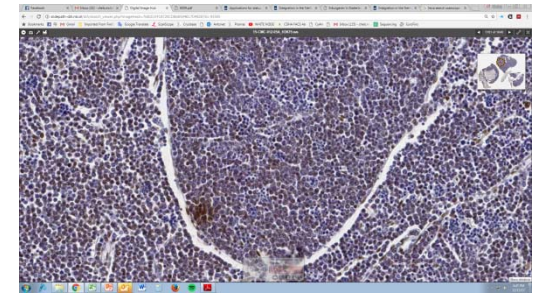

*HDAC1Δ/Δ*  
*DOT1LΔ/Δ*

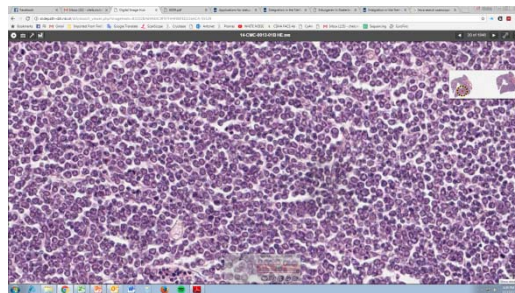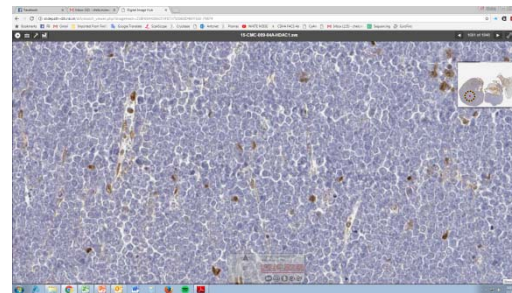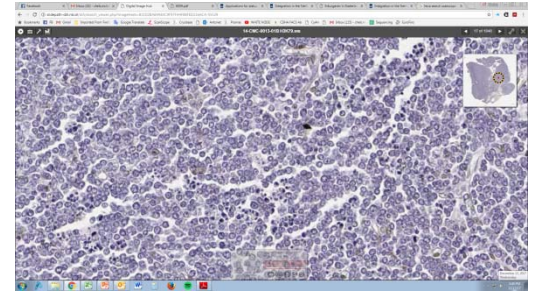

*HDAC1+/+*  
*DOT1LΔ/Δ*

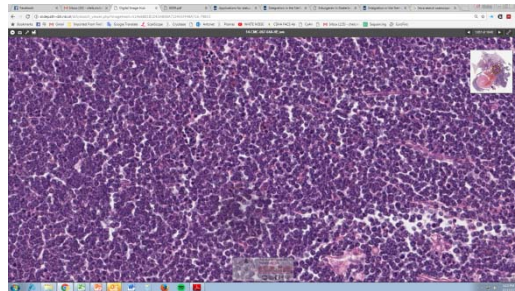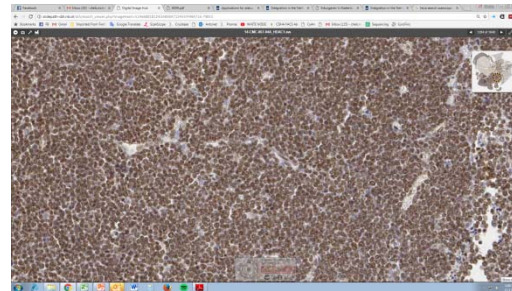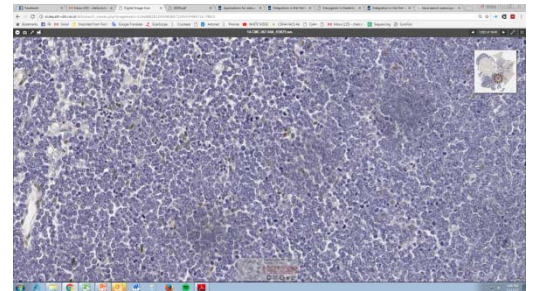

— 10uM  
— 50uM

Supplement: Supplementary file 7 — Source Data for Figure 3 [file EMBJ-38-e101564-s005.zip › embj2019101564-sup-0005-SDataFig3/embj2019101564-sup-0009-SDataFig3B.pdf]

**Fig 4A**

Cell lines:

8995 8996 8999 9002

**HDAC1**

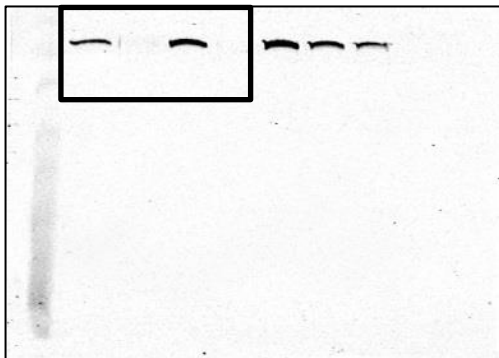

**H3K79me1**

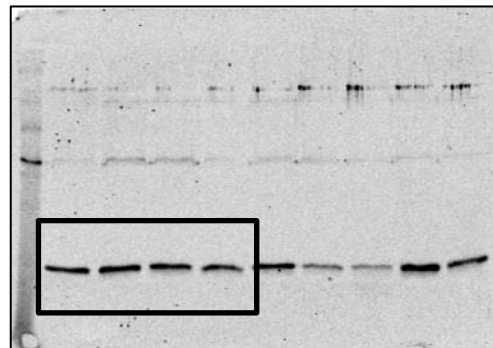

**H3K9ac**

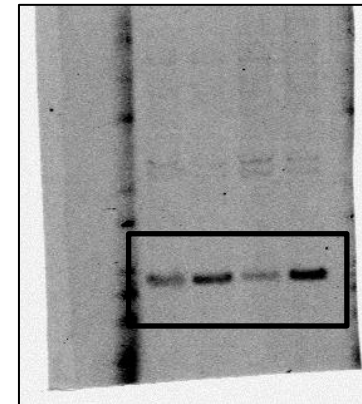

**H3c**

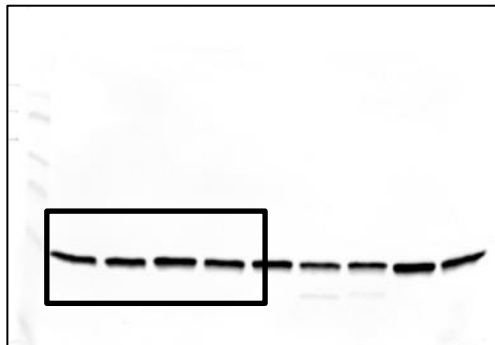

**H3K79me2**

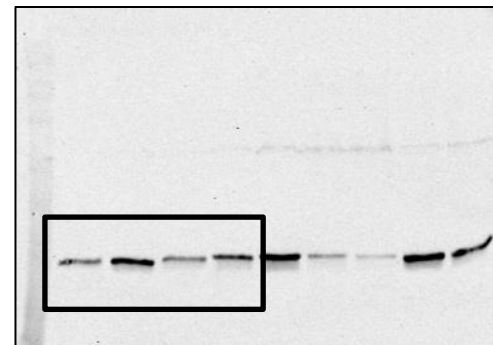

**H4**

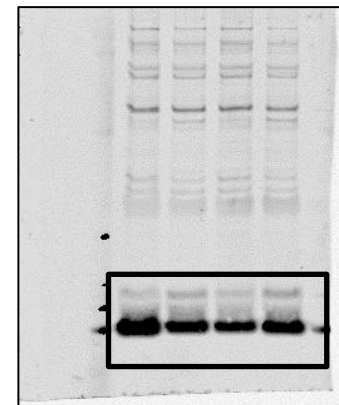

Supplement: Supplementary file 8 — Source Data for Figure 4 [file EMBJ-38-e101564-s006.zip › embj2019101564-sup-0006-SDataFig4/embj2019101564-sup-0011-SDataFig4A.pdf]

**H2BK120ub1**

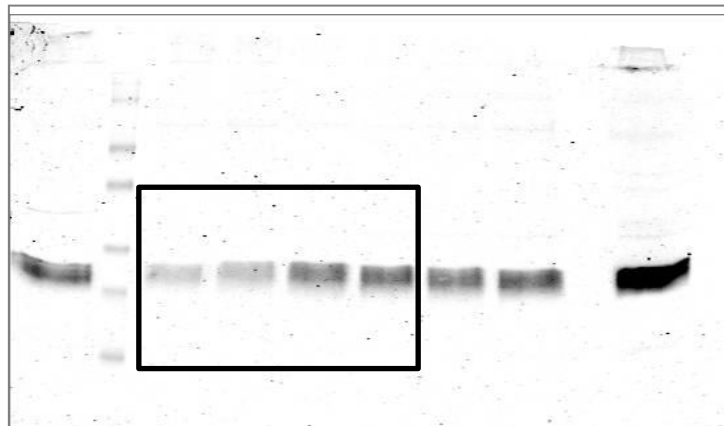

**H3**

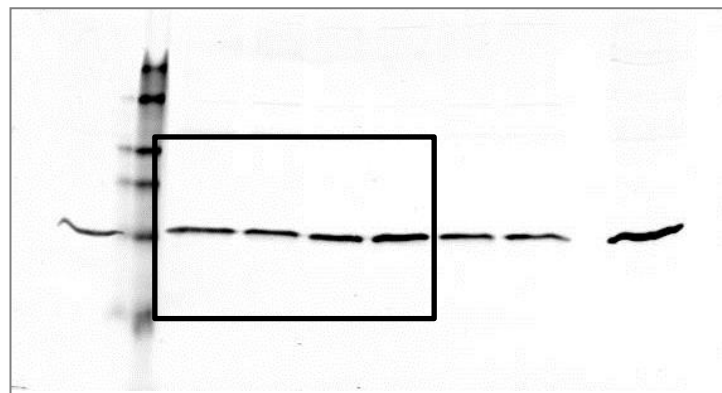

Supplement: Supplementary file 8 — Source Data for Figure 4 [file EMBJ-38-e101564-s006.zip › embj2019101564-sup-0006-SDataFig4/embj2019101564-sup-0013-SDataFig4C.pdf]
